# High-Pressure Structural and Electronic Properties of Bibenzyl (1,2-Diphenylethane) from Synchrotron SC-XRD and Two-Photon-Induced Fluorescence

**DOI:** 10.1021/acs.cgd.5c01569

**Published:** 2026-01-08

**Authors:** Milo Agati, Sebastiano Romi, Samuele Fanetti, Gaston Garbarino, Julien Haines, Roberto Bini

**Affiliations:** † 9300 LENS, European Laboratory for Non-linear Spectroscopy, Via N. Carrara 1, I-50019 Sesto Fiorentino, Firenze ,Italy; ‡ Dipartimento di Chimica “Ugo Schiff”, Università di Firenze, Via della Lastruccia 3, I-50019 Sesto Fiorentino, Italy; ¶ ICCOM-CNR, Istituto di Chimica dei Composti OrganoMetallici, Via Madonna del Piano 10, Sesto Fiorentino I-50019, Firenze ,Italy; § 55553 European Synchrotron Radiation Facility, ESRF, 71 Avenue des Martyrs, CS40220, Cedex 9 38043 Grenoble, France; ∥ ICGM, UMR5253, CNRS, Université de Montpellier, ENSCM, 1919 route de Mende, Cedex 5 34293 Montpellier, France; ⊥ INO−CNR, Istituto Nazionale di Ottica, Via N. Carrara 1, I-50019 Sesto Fiorentino, Firenze, Italy

## Abstract

The structural and electronic properties of bibenzyl (1,2-diphenylethane) are investigated as a function of pressure using single-crystal X-ray diffraction (SC-XRD) and two-photon-induced fluorescence. Bibenzyl is an appealing pseudostilbene compound for the high-pressure synthesis of mixed carbon nanothreads with tunable optical properties, due to its fully saturated linking group. Crystal structures are refined from ambient pressure up to 37 GPa by synchrotron XRD, and the appearance of pseudosymmetry traits at 13 GPa reveals a phase transition that consists of molecular distortions leading to the tripling of the *a* axis and asymmetric unit while maintaining the unit cell's point group symmetry. Two-photon-induced fluorescence up to 18 GPa provides insights into the structural changes where the strengthening of π–π interactions at the phase transition stabilizes the electronic ground state and drives the molecular distortions that occur at that pressure. Coupling structural and electronic properties rationalizes the high-pressure reactivity compared to other pseudostilbenes, showing that the molecular evolution from the added torsional degree of freedom and establishment of π–π interactions at the phase transition lead the molecular conformation to an unfavorable state for pressure-induced polymerization.

## Graphical abstract

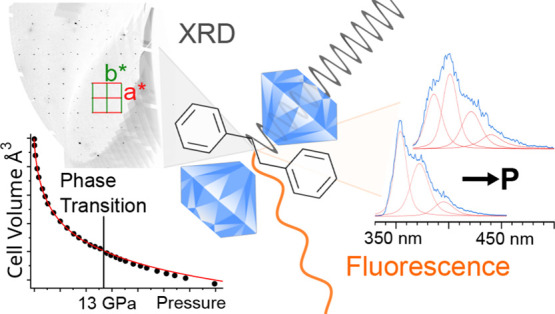

## Introduction

1,2-Diphenylethane, commonly known as bibenzyl, is an aromatic hydrocarbon consisting of two phenyl groups connected by an ethane bridge. Bibenzyl has been recently employed and studied as an organic scintillator for neutron detection, due to its fast scintillation response and inertness of the crystal faces at ambient conditions,^1,2^ and it is also the basis for a variety of organic molecules with a wide range of biological activities such as antitumor,^3,4^ antidiabetic,^5^ neuroprotective,^4–6^ and more. The two phenyl groups permit a wide range of ring functionalizations, and the ethane moiety bridging the two phenyl groups grants a high degree of mobility to the overall structure.^7,8^ Additionally, the fully saturated ethane linking group limits the reactivity to the aromatic rings, apart from benzyl radical formation.^9^ This makes bibenzyl an optimal candidate for the high-pressure polymerization into carbon nanothreads,^10^ as this has been done for similar compounds such as stilbene, azobenzene, and diphenylacetylene, whose high-pressure reactions lead to the synthesis of double-core carbon nanothreads.^11–13^ In fact, bibenzyl has a molecular structure similar to these compounds (Figure 1), and, as such, it is considered part of the pseudostilbene class, with the appealing possibility of forming mixed crystals with these compounds for the synthesis of double-core carbon nanothreads with varying physicochemical properties depending on the nature and number of unsaturated groups connecting the phenyl moieties.^12^ However, bibenzyl presents key differences in its molecular and crystalline structure, which differentiate its high-pressure behavior from those of stilbene and azobenzene. While all of them belong to the monoclinic space group 14 with similar cell parameters,^14–17^ bibenzyl presents a different packing of the molecules in the unit cell (Figure 1). The packing coefficients^18,19^ (*V*_VdW_/*V*_cell_) for azobenzene and stilbene at ambient conditions are 67.1 and 67.2%, respectively, while bibenzyl has a packing coefficient of 64.5%. Therefore, azobenzene and stilbene have a more efficient packing of the molecules similar to a hexagonal packing, whereas in the bibenzyl unit cell, the molecules are disposed parallel along a specific direction, resembling more a cubic packing. Furthermore, the bibenzyl molecule is not planar, and it has a much greater degree of freedom for rotation around the ethane linking group than stilbene and azobenzene, which instead, in the crystalline structure, are limited only to a pedal motion.^20^ These differences have been identified as the main cause for the lower reactivity of bibenzyl at high pressure.^21^

**Figure 1:**
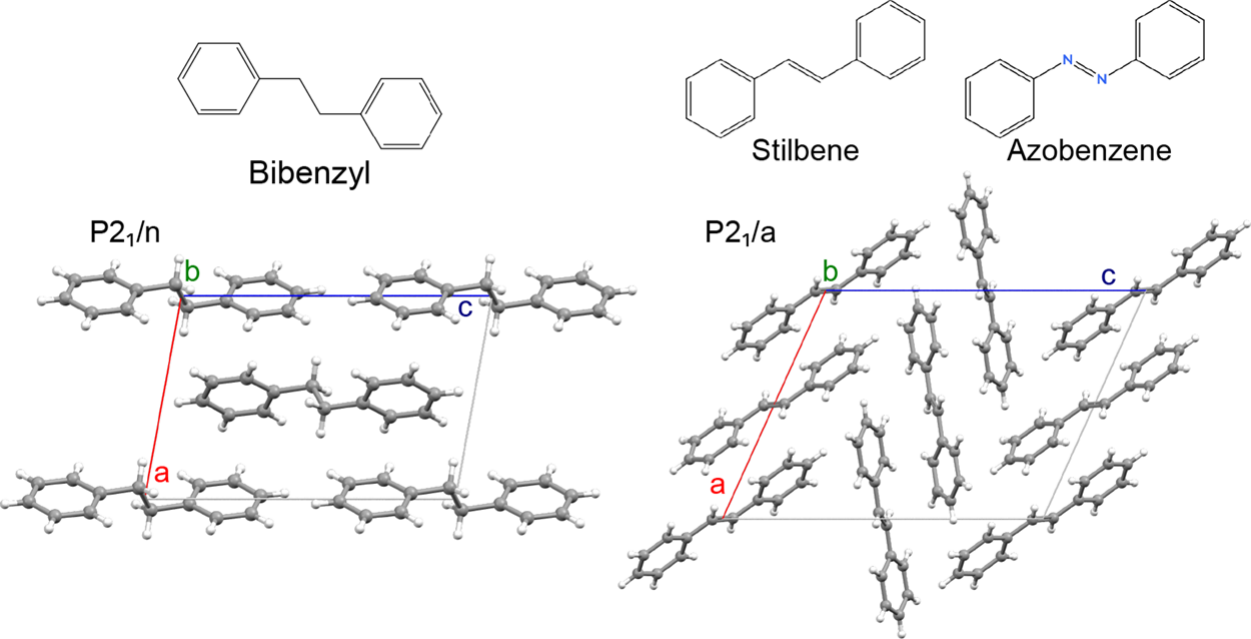
Structural formula and projection along the *b*-axis of the crystalline structure of bibenzyl (left) and stilbene/azobenzene (right).

Another important difference with regard to the aforementioned members of the pseudostilbene class is the absence of π bonds between the phenyl groups and the presence of nodes on the ethane moiety in the molecular frontier orbitals. As a result, electronic delocalization across the entire molecule is suppressed, and the first electronic excited state is of ππ* nature localized on the individual aromatic rings.^2,22^ This is reflected in the UV–vis absorption and emission spectra of the monomer, where the S_0_ → S_1_ and S_1_ → S_0_ transitions are at higher energies than other conjugated diphenyl compounds such as stilbene.^23,24^ In the solid state, the intermolecular interactions do not influence the ground state given that the absorption is still peaked at the same wavelength as that of the isolated molecule.^22^ In contrast, these interactions remarkably affect the excited state as it is attested by the emission spectra, which presents a broad emission band red-shifted by about 5000 cm^–1^.^25–27^ This is reported for many aromatic compounds due to the emission from excimers, but usually their formation leads to emission quenching by extended delocalization through π–π interactions.^28,29^ Conversely, in the bibenzyl case, the emission derived from the excimeric species is not quenched, and it is observed to have a greater quantum yield than the monomer emission. This phenomenon has been labeled as “aggregation-induced emission,” and it is the opposite of the aggregation-caused quenching.^22,30^ This is straight evidence that fluorescence directly probes the intermolecular interactions and can be used to rationalize the structural data obtained from single-crystal X-ray diffraction (SC-XRD).

Here, we provide a comprehensive study of the structural evolution of a bibenzyl crystal under high pressures, motivated by its appealing employment as a spacer in mixed nanothreads from pseudostilbenes and aimed at understanding the molecular mechanisms responsible for the different reactivities compared to other pseudostilbenes. SC-XRD using synchrotron light provides direct information on the structural evolution in compression up to 37 GPa. The complete structural information provided by this technique is coupled with the measurement of the bibenzyl fluorescence as a function of pressure. The fluorescence probes the electronic states of a compound and can provide valuable information on the intermolecular interactions present in a crystal and on the changes occurring as pressure increases.^31–34^

## Results and Discussion

### Ambient-Pressure Crystal Structure

The ambient-pressure crystal structure of bibenzyl has been determined from SC-XRD measurements with synchrotron light as well as with an in-house diffractometer. The crystal measured with in-house instrumentation was mounted on a transparent microloop under a flux of cold nitrogen gas (173 K); this way, thermal motion is reduced, leading to sharper peaks and more precise structural determination.^35^ The use of a transparent microloop mount allowed for an almost complete collection of reflections (97% completeness), as opposed to the crystal measured with synchrotron light, which was loaded at ambient conditions inside a diamond anvil cell (DAC), which shadows the majority of the reciprocal space (39% completeness). Structure refinement from both crystals leads to nearly identical results aside from the differences arising from the measurement temperature. See Tables S1 and S2 for the meaningful measurement parameters and refinement data.

The unit cell belongs to the monoclinic space group no. 14 *P*2_1_/*n*,^17^ and the crystal structure has been refined with *Z* = 2 (asymmetric unit C_7_H_7_) with the molecules lying on Wyckoff sites 2a (0, 0, 0, and 1/2, 1/2, 1/2; site symmetry: -1). The unit cell packing and molecule labeling are shown in Figure S1. The point group symmetry of the bibenzyl molecule is *C*_*i*_, as opposed to the previous association to the *C*_2*h*_ space group^17,36^ ; the two phenyl groups lie parallel on two different planes, and the torsional angle (C2C1C7C7*) is equal to 73.4(5)° at ambient conditions and 68.51(8)° at 173 K, therefore deviating from a *C*_2*h*_ point group symmetry, which would have required a 90° torsional angle.^37^ The presence of two distinct conformers in the crystal deriving from the pedal motion of the molecule^17^ can be excluded given that the highest residual electron density calculated is equal to only 0.065 e^–^/Å^3^, too low to indicate the presence of another minor conformer. At 173 K, a conformational disorder is not expected, and the two highest residual electron densities are equal to ∼0.2 e^–^/Å^3^ corresponding to bonding electrons of the ethane moiety and to the relative librating methylene hydrogen atoms.^17^ Lower residual electron density does not form any meaningful structure.

Nonetheless, the equivalent isotropic displacement parameters (*U*_eq_) have high values at ambient temperature with the average between all carbons equal to 0.105(5) Å^2^ (Figure 2); this indicates the presence of dynamic disorder, which derives from low-frequency molecular vibrations from torsional movements.^17,21^ The disorder leads to the calculation of a shorter ethane bond, equal to 1.484(6) Å in our refined structure at ambient conditions, in agreement with previous reports.^17,37,38^ This is confirmed by the low-temperature structure at 173 K, which presents a regular ethane bond length of 1.5231(14) Å and much lower *U*_eq_s with the average between all carbons equal to 0.034 Å^2^.^17,37^ A similar effect is observed with increasing pressure, as the *U*_eq_s present a substantial reduction, reaching an average minimum value of 0.024(3) Å^2^ at about 5 GPa (Figure 2). This behavior is expected as molecular motion is constrained by the significant density increase.^39,40^ As such, the ethane bond length value behaves in accordance with increasing pressure, reaching between 1 and 2 GPa the same values calculated in the low-temperature structure (Figure S2).

**Figure 2:**
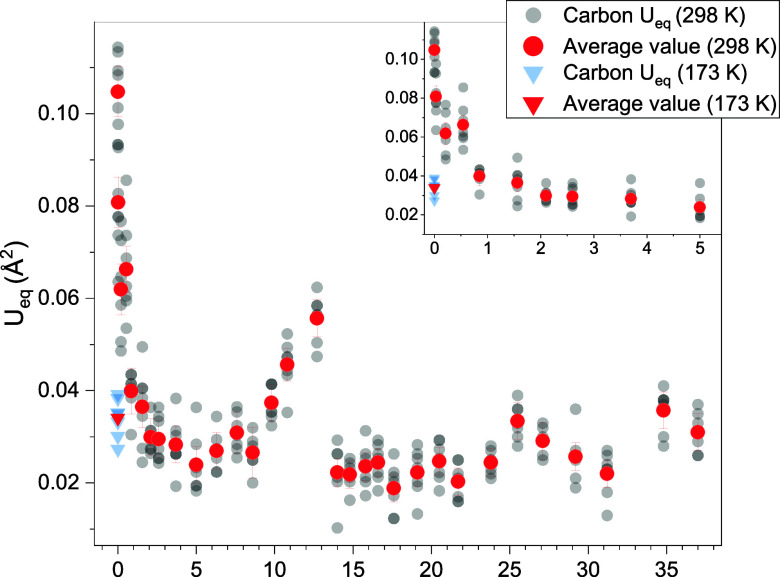
Pressure evolution of the equivalent isotropic displacement parameters (*U*_eq_). The semitransparent gray dots are related to each carbon atom for every pressure point, whereas the red dots are the average values between all of the carbon atoms. Downward pointing triangles characterize the isotropic displacement parameters of the structure refined with in-house instrumentation at 173 K. Only the error bars for the average values are reported for visual clarity. The inset shows a magnified portion of the graph from 0 to 5 GPa to better visualize the rapid decrease of the *U*_eq_ values as pressure is applied. The decrease up to 5.0 GPa is ascribed to a reduction of molecular motion caused by the increased crystal density, whereas higher values from 5.0 to 12.7 GPa are due to an increase in static disorder in the crystal. After the phase transition at 13 GPa, the accumulated stress is released with structural changes that greatly reduce the magnitude of the static disorder and thus the *U*_eq_ values. The scattered values calculated above 22 GPa arise from a progressive quality loss of the crystal.

### High-Pressure Data

Bibenzyl (1,2-diphenylethane) single crystals were compressed in a DAC up to 37 GPa using helium as the pressure-transmitting medium (PTM), which ensured quasi-hydrostatic conditions up to the maximum pressure.^41^ All of the measurements were collected at ambient temperature (23 °C ± 1 °C). Synchrotron X-ray radiation is employed, and diffraction data of two bibenzyl crystals were collected (approximate size 20 × 20 × 10 μm). The image of the sample chamber after gas-loading is displayed in Figure S3. One of the two loaded crystals remained intact for the majority of the compression, and its orientation was favorable for the collection of sufficient data for the refinement of a crystalline structure up to 37 GPa. The unit cell parameters and volume evolution as a function of pressure are displayed in Figure 3. The evolution of the *P*2_1_/*n* unit cell parameters shows a clear discontinuity around 13 GPa, highlighting a phase transition and therefore defining low-pressure and high-pressure phases. The changes observed up to this pressure value closely resemble the changes previously observed for a powder sample compressed without a PTM (Figure S4).^21^ A greater compressibility from ambient pressure to 13 GPa is observed along the *a*-axis, which reduces by 20%, as opposed to the *b* and *c* parameters, both of which reduce by only ∼ 10%. The monoclinic β-angle shows a significant increase of 10°. The cell volume data up to 13 GPa have been fitted by a third-order Rose–Vinet equation of state^42,43^ :

$$P=3\times B_{0}\frac{1-{\left(\frac{V}{V_{0}}\right)}^{1/3}}{{\left(\frac{V}{V_{0}}\right)}^{2/3}}\times \exp\left[\frac{3}{2}\times (C_{0}-1)\times \left(1-{\left(\frac{V}{V_{0}}\right)}^{1/3}\right)\right]$$ 1

where *V*_0_ is the cell volume at ambient pressure, while *B*_0_ and *C*_0_ are respectively the isothermal bulk modulus and its derivative with respect to pressure at *P* = 0. The calculated values obtained for *V*_0_, *B*_0_, and *C*_0_ by the fit are in perfect agreement with the literature data^17^ and with the powder compression without PTM,^21^ indicating an identical evolution of the unit cell up to the phase transition in non- and quasi-hydrostatic compression conditions.

**Figure 3:**
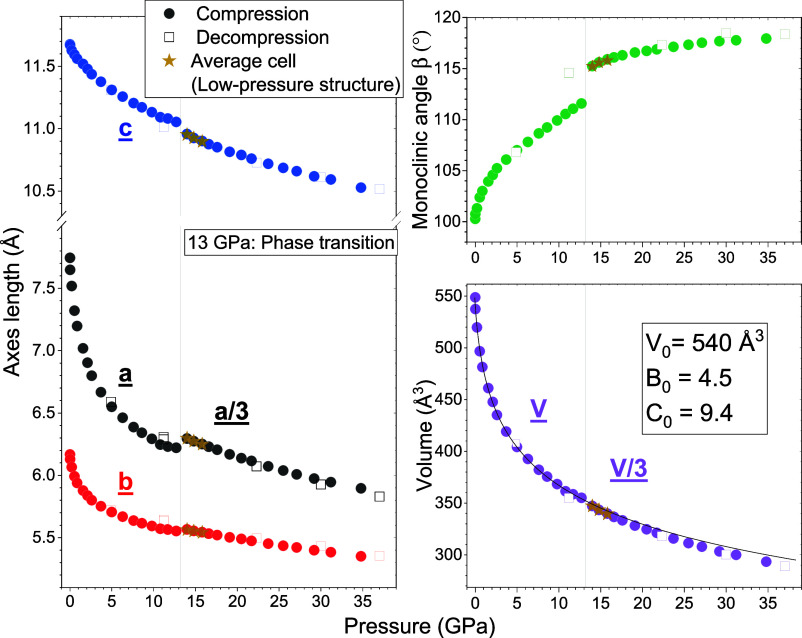
Evolution as a function of pressure of the unit cell parameters (left), monoclinic angle (top right), and volume (bottom right) of bibenzyl. Filled dots represent the measurements taken in compression, while empty squares represent the data in decompression. The gold star-shaped symbols refer to structures refined without the satellite reflections appearing after the phase transition. After the phase transition, the value of the *a* parameter triples; therefore, in the graph is reported 1/3 of the value for continuity with the low-pressure phase. The same is also done for the volume. Error bars smaller than the symbol size characterize the data.

The phase transition occurring at 13 GPa is highlighted by the appearance of pseudosymmetry traits in the recorded diffraction pattern. Many new reflections appear (Figure 4) which can be perfectly accounted for with the addition of the modulation vector (0.333, 0, 0) to the same *P*2_1_/*n* unit cell of the low-pressure phase. This means that along the a* axis in the reciprocal space, two new equally spaced reflections appear between reflections of the low-pressure phase. The totality of the reflections can be indexed as a whole, and their intensities and systematic absences lead to the same *P*2_1_/*n* unit cell although with triple the *a* cell parameter value. As such, in Figure 3, one-third of the *a* parameter and volume values are reported in the graphs for better description of the unit cell evolution.

**Figure 4:**
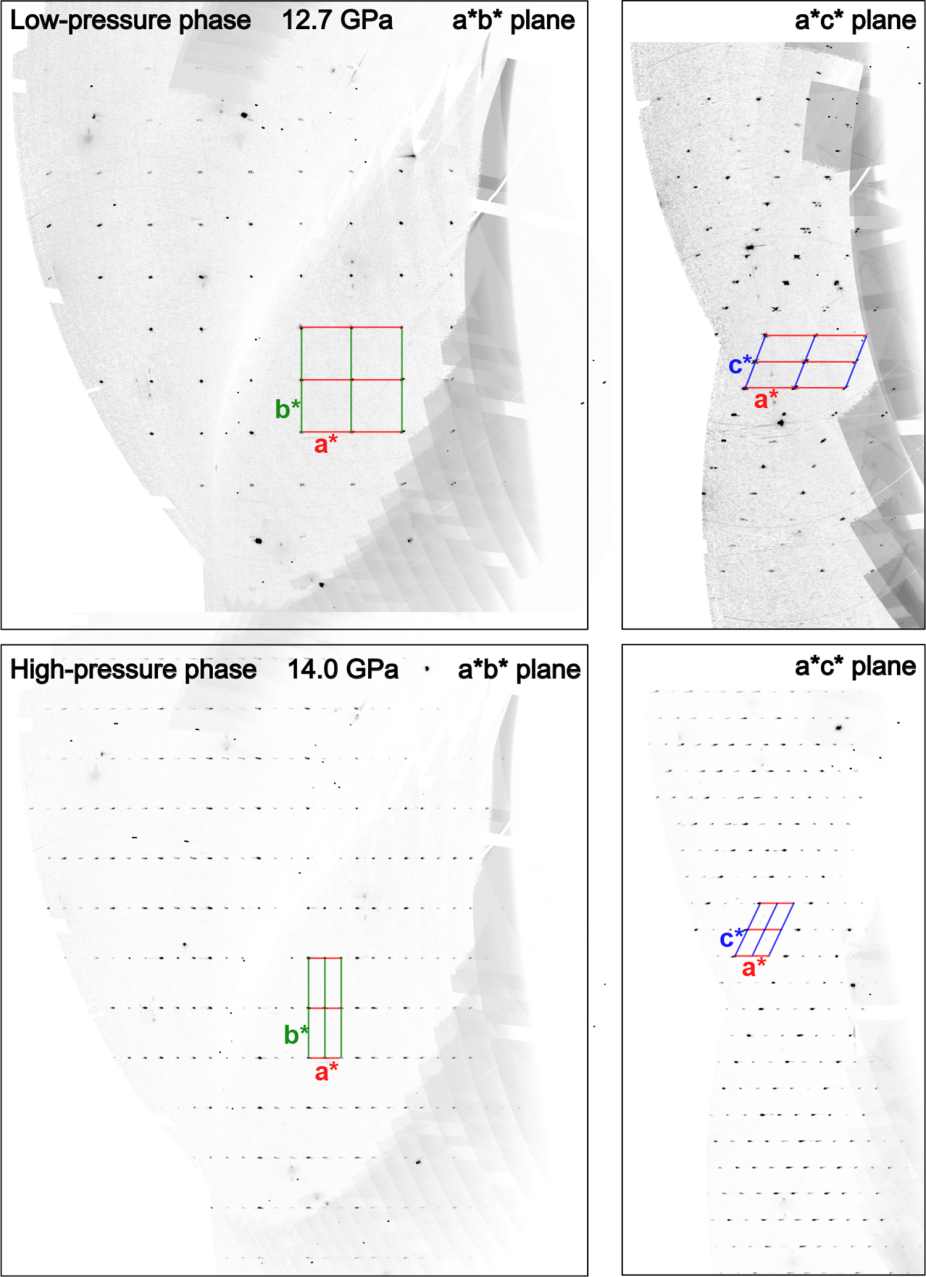
Reciprocal space view projections onto the a*b* (left) and a*c* (right) planes before (top panels) and after (bottom panels) the phase transition. The reciprocal-lattice grid is overlaid on the images. After the phase transition, new periodic reflections appear between those of the low-pressure phase along the a* axis. The new reflections are perfectly described by a single modulation vector *q* = (0.333, 0, 0), while the reflections of the low-pressure phase remain almost unaltered during the phase transition.

The high-pressure phase unit cell is displayed in Figure 5. This reveals that the tripling of the *a*-axis is directly related to the tripling of the asymmetric unit contents, passing from half a molecule positioned on an inversion center (formula C_14_H_14_; *Z* = 2; *Z*′=0.5) to the same unit plus an additional symmetry nonequivalent molecule (formula C_14_H_14_; *Z* = 6; *Z*′=1.5). In Figure S5, the atom labeling employed for this new structure is shown. This could be related to a *k-type* phase transition^44^ with a reduction of the translational symmetry caused by distortions of some molecules from the applied stress; more precisely, it is as if 2 of every 3 layers of molecules (one unit cell) in the *bc* plane become distorted in pairs.

**Figure 5:**
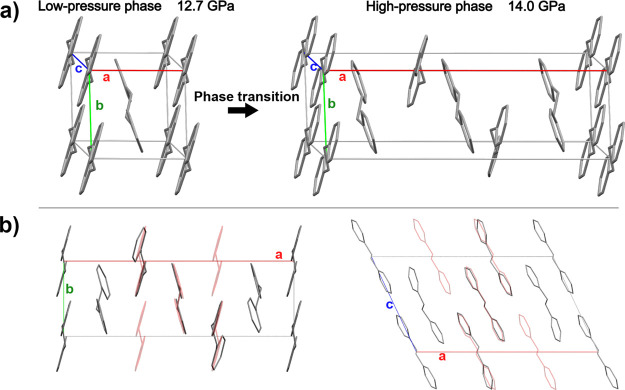
(a) Low-pressure crystal structure of bibenzyl (left) and high-pressure structure (right). Both structures are *P*2_1_/*n*; however, after the phase transition, a tripling of the unit cell along the *a* axis occurs. This derives from a reduction in symmetry caused by a distortion from the applied high pressure. The asymmetric unit also triplicates in atomic content becoming two non-symmetry-equivalent bibenzyl molecules, with one of them positioned on an inversion center (Figure S5). (b) Projections along the *c* and *b* axes of the high-pressure structure. An overlay of the structure refined without satellite reflections at the same pressure is drawn in red to better highlight the tripling of the unit cell and the molecular distortions.

At the phase transition, in addition to the observed molecular distortions, all of the cell parameters show appreciable discontinuities in their pressure evolution (Figure 3). The *b-*cell parameter increases very slightly from 5.553 Å at 12.7 GPa to 5.566 at 14 GPa, while the *c* parameter decreases a bit more from 11.054 to 10.957 Å. Considering one-third of the *a* parameter value, it increases from 6.221 to 6.295 Å (18.884 Å refined value), and the monoclinic angle β increases by ∼4°, as similarly observed for azobenzene and stilbene.^45,46^ The unit cell volume does not present an abrupt reduction but only a slight increase in its compressibility. These small changes are in agreement with the appearance of distorted structures without major molecular rearrangement, as opposed to the phase transition of azobenzene, which exhibits abrupt changes in the cell parameters caused by consistent molecular rearrangement.^46^ Data have also been acquired in decompression, revealing a complete reversibility of the phase transition. The cell parameters in decompression (empty square symbols in Figure 3), although with some hysteresis, perfectly overlap the compression data. In addition to this, all of the satellite reflections disappear after the phase-transition pressure. The crystal structure in decompression could not be refined due to a drastic decrease in crystal quality from pressure release.

The reduction of the unit cell volume involves significant changes in the molecular structure. Some useful quantities to better visualize the molecular changes occurring during compression are displayed in Figure 6. A torsional movement is observed, and as shown by its evolution as a function of pressure in Figure 6a, the associated torsion angle (C2C1C7C7*) increases up to about 5 GPa. The effects of this molecular rearrangement are multiple; there is a tendency toward coplanarization of the phenyl groups of the single molecule, as shown by the interplanar distance (Figure 6e), which decreases from 1.2 Å to a minimum of 0.5 Å. From the obtained structures, it is possible to observe that perfect coplanarization is not achieved even after the phase transition. Together with the torsional movement, a rotation of the whole molecule occurs, which brings the molecules nearly perpendicular to the *a* axis of the cell. This is in accordance with the larger reduction of the *a* parameter, which is the preferential direction of compression due to the free space available along this direction, in contrast to the *c* direction, where the molecules are aligned along their length. This also explains the strong directionality observed in the anisotropic displacement parameters (ADP) along the *a* axis direction with a much greater diagonal term relative to the *a* axis (*U*_11_) with respect to those relative to the *b* (*U*_22_) and *c* (*U*_33_) axes (Figure S6). The distance between the rings' centroids exhibits an interesting evolution as it first slightly increases to then decrease constantly after 1 GPa (Figure 6d). This is reflected by the *c* axis evolution, which shows a very small discontinuity at 1 GPa, followed by a compression along the length of the molecules that point against each other along the *c*-axis direction. The same cell parameter behavior was observed in the compression of a powder sample having a counterpart in the observed changes of the internal and external IR and Raman modes.^21^

**Figure 6:**
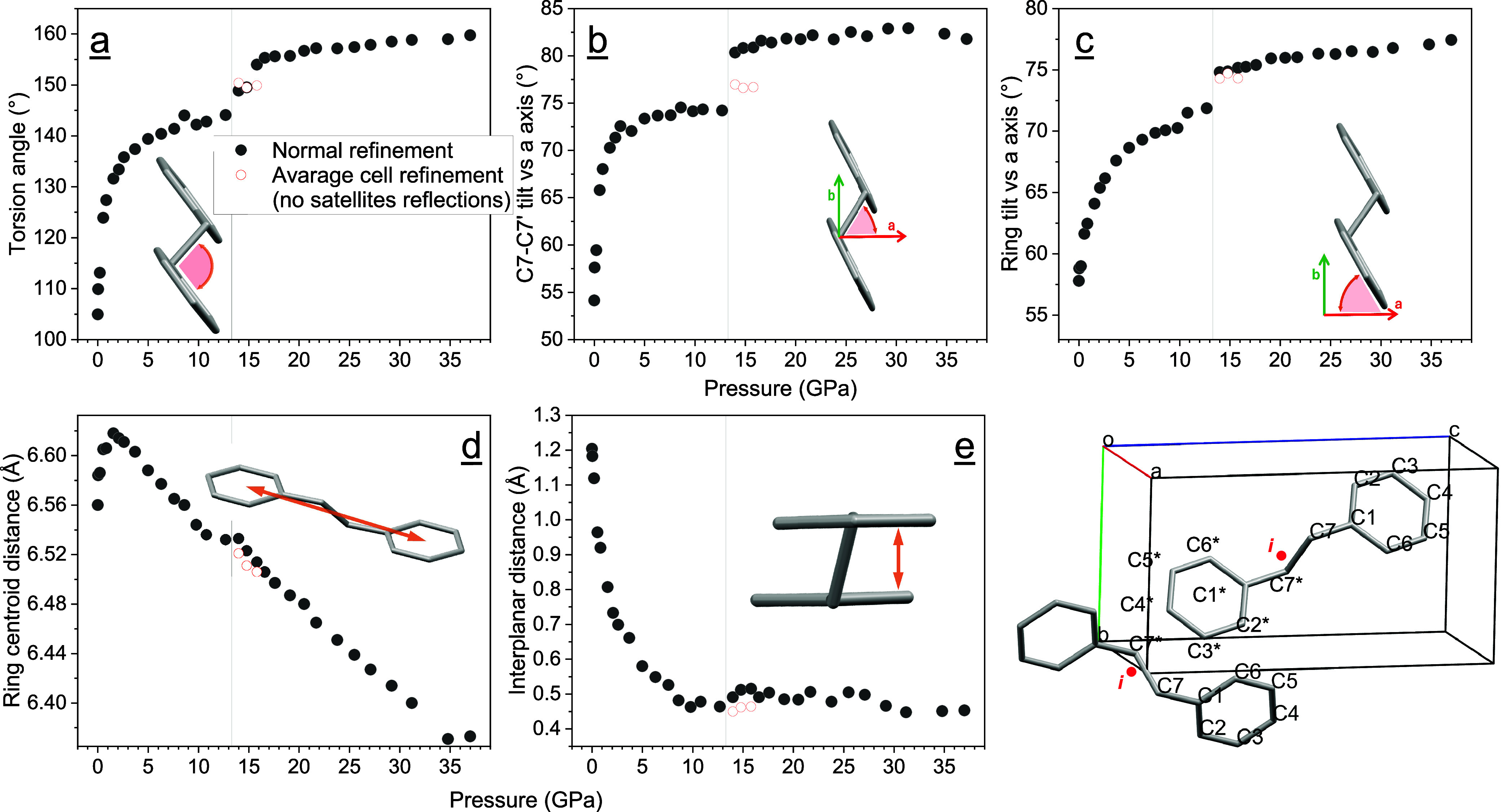
Significant molecular parameters (angles and distances) as a function of pressure. In the bottom right, a representation of the unit cell with the employed labeling is shown. (a–e): Torsion angle (C2C1C7C7*); ethane moiety tilt (C7C7*) with respect to the *a* axis; phenyl group tilt with respect to the *a* axis; distance between phenyl groups of the same molecule; and interplanar distance of the phenyl groups of the same molecule. The black dots refer to the bibenzyl molecule with the *C*_*i*_ point group symmetry residing in the *2a* Wyckoff position in both the low- and high-pressure phases, while the empty red dots refer to the same molecule but for the average structure cell refined without the satellite reflections appearing after the phase transition at 13 GPa. Errors are within the symbol size.

While the phase transition mainly consists of the appearance of lower-symmetry molecular structures, the molecules that lie on the inversion centers (Wyckoff position *2a*) maintain their point group symmetry and undergo sudden changes in their structure. In Figure 6a–c, the torsional angle increases by ∼15° after having reached a plateau before the phase transition, and the entire molecule rotates to lie more parallel to the *b* axis (perpendicular to the *a* axis), as shown by the tilt angle of the ethane bond and the phenyl groups with respect to the *a* axis. In addition, the equivalent isotropic displacement parameters of these molecules (Figure 2), as previously stated, decrease with increasing pressure, reaching a minimum around 5.0 GPa due to the reduced molecular motion and dynamic disorder from the density increase. With further increase in pressure, the *U*_eq_s increase again, reaching a maximum average value of 0.056(4) Å^2^ at 12.7 GPa, just below the phase-transition pressure. An increase in the dynamic disorder is excluded as the molecular motion is highly constrained at these pressure values. Therefore, it is ascribed to a growth of static disorder upon compression. After the phase transition at 14.0 GPa, the *U*_eq_s abruptly decrease, returning near their average minimum value at 0.023(3) Å^2^. This sudden reduction of static disorder is related to the release, at the phase transition, of the stress that accumulated during the compression. With further increase in pressure, the *U*_eq_ values remain constant, while above 22 GPa, scattered values are calculated. This is due to a progressive decrease in the crystal quality, leading to a lower data/parameter ratio and thus to larger displacement parameters. A similar behavior was previously observed in the pressure evolution of the displacement parameters of azobenzene as well as its pedal motion disorder with increasing pressure.^46^

Crystal structures excluding the satellite reflections (Table S3) could be refined only up to 17 GPa, thus obtaining the unit cell of the average structure.^47^ The refined average cell is nearly identical to the unit cell before the phase transition, and we observe the same discontinuities in the cell parameters with values identical to those of the complete structure (see the gold star-shaped symbols in Figure 3). This is to be expected, and it is in excellent agreement with the fact that the obtained average unit cell describes the average structure of the one refined with all the reflections. However, the molecular changes observed with this structure still consist of an increase in the torsional angle and tilt of the molecule, which are in contradiction to an increase along the *a*-axis. Additionally, the ADPs of the carbon atoms in the average structure are around double the value of carbon atoms in the complete structure (Figure S7), given that molecular distortions have to be taken into account.^47^ Therefore, the refined average structure is not fully representative of the material after the phase transition, and thus, we will continue using the complete structure rather than the average structure, as it will also make sense from the Raman and fluorescence measurements.

Additional information is retrieved by analysis of the integrated diffractograms (Figure 7) and the sum of images of the diffraction patterns (Figure S8). At the phase transition at 14 GPa, the appearance of new reflections throughout the integrated pattern and abrupt discontinuities in the pressure shift of all of the peaks are evident, as shown in Figure 7. The new peaks are ascribed to the satellite reflections appearing at the phase transition, which undergo minimal changes in their intensities up to 37 GPa. The two peaks appearing at 1.16 and 1.27 Å^–1^ can be related to the peaks observed in a powder compression^21^ (1.14 and 1.34 Å^–1^) around the phase-transition pressure. Another peak at 3.9 Å^–1^ is easily discernible after the phase transition, and it stands out for its remarkable shift and intensification with pressure. This peak appears in the integrated image as a faint diffraction ring with bright spots, characteristic of an oriented powder, which becomes more intense with increasing pressure (Figure S8). After 24 h at 35 GPa, the bright spots are much more defined and their disposition in an octagonal geometry is more evident. This type of diffraction ring was previously observed in the pattern of the recovered material from the compression of a bibenzyl powder.^21^ At 24 GPa, which is a typical pressure threshold for similar aromatic compounds for the reaction into carbon nanothreads, another reflection appears at 0.98 Å^–1^, corresponding to 6.33 Å of interplanar spacing. This reflection has a negligible pressure shift and its *d*-spacing value is similar to the characteristic reflection found in the recovered pseudostilbenes after high-pressure reaction into carbon nanothreads.^12,13,21,45^ While signs of a reaction are not observed, this new peak could be related to a prereactive arrangement of the molecules. Additional faint diffraction rings between 2.4 and 2.8 Å^–1^ are observed after the phase transition; however, in decompression, all of these features, together with the satellite reflections, completely disappear. Therefore, all of the features observed at high pressure do not derive from an unrecoverable loss of crystallinity due to the high stress but rather from reversible distortions or even from a new phase. The disappearance of the powder-like reflections in decompression is not ascribed to amorphization as no clear signs of the formation of an amorphous material are observed. The diffractograms without the background component removed (Figure S9) maintain their overall profile both in compression and in decompression, not showing any sign that suggests the formation of an amorphous material in bulk.

**Figure 7:**
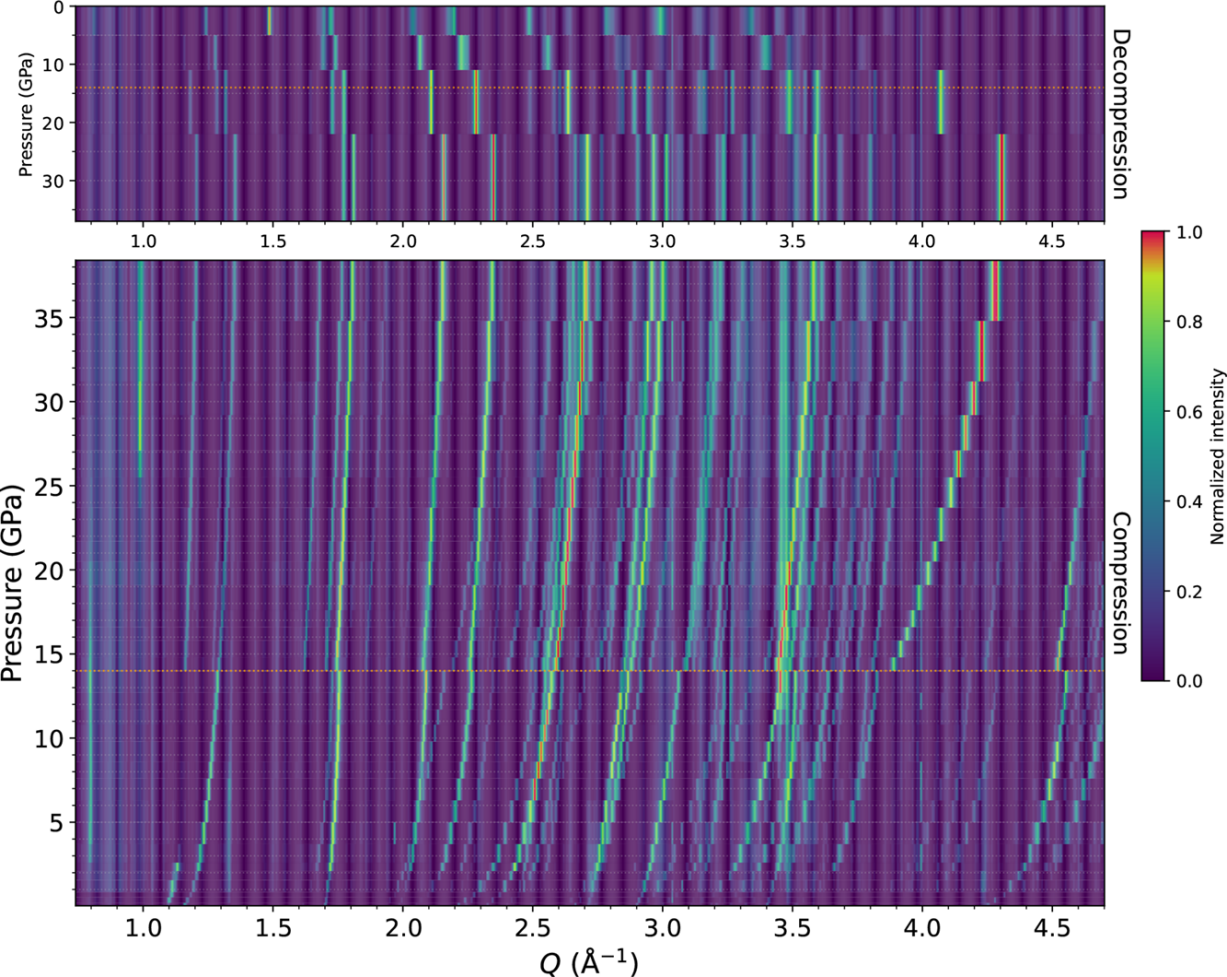
Color map of the normalized azimuthally integrated diffraction patterns in compression and decompression (top graph). To improve the visualization of weaker and stronger features, the data are log-scaled, normalized, and subjected to a gamma correction, accentuating higher intensity peaks relative to the baseline. New peaks appear at the phase transition and at 24 GPa, all of which disappear, crossing back the phase-transition pressure in decompression. An orange line highlights the phase-transition pressure.

However, no new reflections that can be ascribed to the formation of a crystalline product are observed at the lowest pressure measured in decompression (5 GPa). Nonetheless, bibenzyl already showed low reactivity at these pressure values without any PTM,^21^ which could have been further reduced by the quasi-hydrostatic pressure conditions. Penetration of helium atoms inside the crystalline structure, blocking the aromatic rings from reacting, can be excluded for multiple reasons. The first one is that the inclusion of helium inside the crystalline structure would likely reduce its compressibility^48,49^ ; however, after the phase transition, the unit cell becomes more compressible with respect to the fitted Rose–Vinet equation of state.^42,43^ Second, from calculations with dedicated software,^19,50^ no solvent accessible voids equal to or greater than 1.4 Å (van der Waals radius of a helium atom) are present. At ambient pressure, the pore limiting diameter (largest sphere that can percolate through the void network) is equal to 1 Å, and the maximum pore diameter (largest void sphere that can fit in the structure) instead is 1.4 Å. With increasing pressure, these values significantly reduce, and at the phase transition, no considerable changes are observed.

### TPA-Induced Fluorescence

Crystalline bibenzyl from Sigma-Aldrich (lot no. STBH6120, purity 99.8%) without any further purification was dissolved in methanol at increasing concentrations (0.005 through 0.5 M). Emissions of the solutions were induced by two-photon absorption (TPA) using a 530 nm excitation wavelength with a maximum pulse energy of 100 nJ and 30 ps pulse duration. Fluorescence spectra were measured with a 1 nm resolution and are displayed in Figure 8a. At the lowest concentration measured, the fluorescence consists of a single asymmetric band centered around 280 nm, and it is directly ascribed to the monomer's emission, mirroring the absorption band at 260 nm due to the excitation to the S1 state with a Stoke shift of ∼ 3300 cm^–1^. With increasing concentration, the monomer's emission intensity decreases in favor of another broad band centered at 355 nm (Figure 8a). This emission that spans from 340 to 450 nm originates from the formation of excimeric species.^22,30^ Moreover, excimer emission is not present at very low concentrations, showing that intramolecular excimers between aromatic rings do not form, but only excimeric species from intermolecular interactions.^51,52^ Two peaks centered at 332 and 348 nm can be distinguished in the emissions of the 50 and 100 mM solutions, which defines a vibronic succession based on the aromatic C=C stretchings with frequencies of ∼1000 cm^–1^. For high concentrations (500 mM), the high-energy side of the excimer emission is cutoff by reabsorption from a secondary band between 280 and 330 nm, which could be related to dispersed crystalline bibenzyl in solution or the presence of a small amount of stilbene impurities (<0.5%, see Figure S10). Note that the fluorescence collection with backward geometry greatly reduces reabsorption, which becomes much more noticeable only at the highest concentrations. Bibenzyl purified via recrystallization on a cold finger from vacuum sublimation and from slow evaporation of an ethanol solution yielded UV–vis spectra nearly identical to those of the untreated batch. In the crystalline state, at ambient pressure, reabsorption still occurs; however, it is also ascribable to absorption from bibenzyl as the absorption edge of pure bibenzyl still extends down to 340 nm.^2,22,53,54^

**Figure 8:**
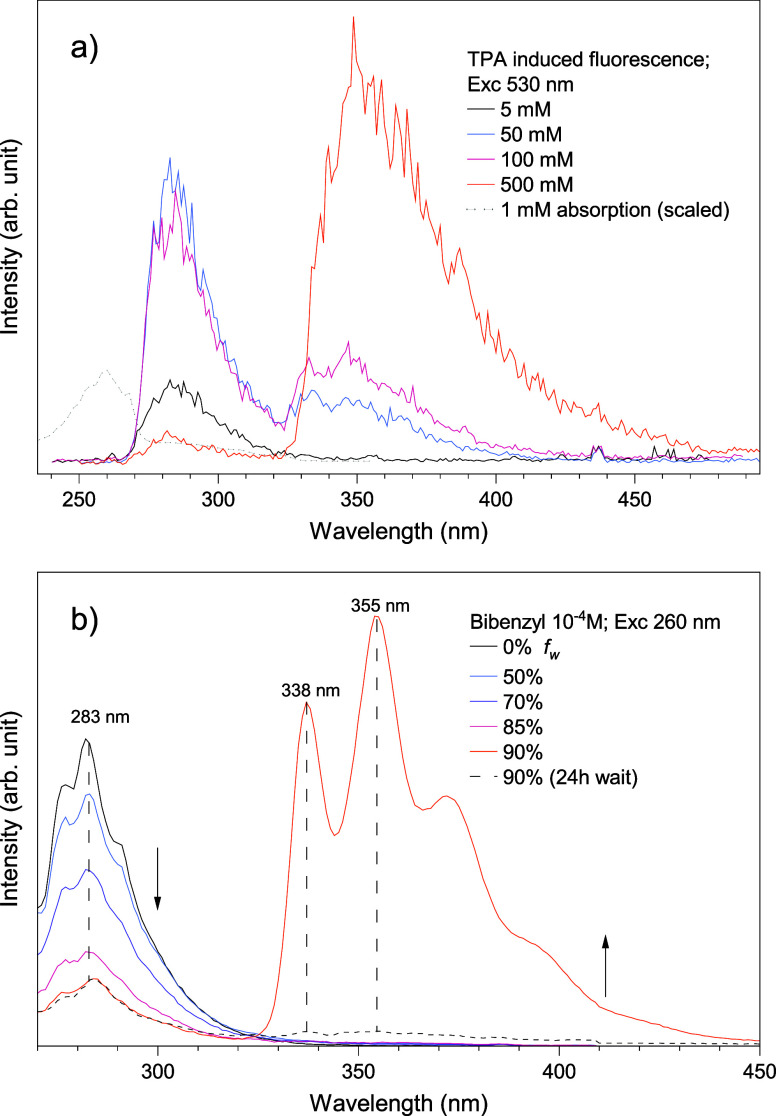
(a) Fluorescence spectra of bibenzyl solutions in methanol with increasing concentration. The absorption spectrum is scaled and overlaid on the image for reference as a dashed trace. The excitation was induced with TPA with a 530 nm excitation wavelength, corresponding to a one-photon absorption (OPA) at 265 nm. The highest energy side of the excimer emission gets cutoff by reabsorption, which becomes considerable at high concentrations as well as in the solid crystal. (b) Water fraction (*f*_w_) graphs of 0.1 mM bibenzyl solutions in ethanol measured on an FLS1000 photoluminescence spectrometer from Edinburgh Instruments. A decrease in the monomer emission is observed upon increasing the water fraction, whereas at 90% *f*_w_, the aggregation-induced emission is observed. After a 24 h wait, the emission from the aggregates is much more reduced while the monomer emission remains unchanged.

To ensure that the measured fluorescence at 355 nm is an aggregate-induced emission (AIE) from the excimer species formed,^22,30^ one-photon-induced fluorescence spectra of bibenzyl solutions with increasingly higher water fractions were measured on a commercially available photoluminescence spectrometer (Edinburgh Instruments FLS1000) and are displayed in Figure 8b. Spectroscopy-grade water fractions were added to bibenzyl solutions in ethanol while maintaining a 0.1 mM final concentration for every measurement. With higher water fractions, a decrease in the monomer emission at 283 nm is observed, and it becomes much more drastic above 70% *f*_w_. At 90% *f*_w_, the structured emission peaked at 355 nm appears, and it is extremely intense while the monomer emission weakens but it is still clearly visible. The excimeric origin of this emission from aggregation is further reinforced by the absorption spectra of the same samples, which do not show any changes with increasing water fractions, aside from a slight broadening at 90% *f*_w_ (Figure S11). After a 24 h wait, the 90% *f*_w_ was decanted and measured again: while the lower energy emission greatly reduces in intensity, the monomer emission remains unchanged. This is a clear indication that the lower energy emission originates from bibenzyl aggregates that precipitate over time.

Fluorescence from crystalline bibenzyl was measured at ambient pressure outside of the DAC and in compression after being loaded without any PTM into the cell. A stainless steel gasket drilled to an initial diameter of 150 μm and a thickness of about 50 μm was employed to contain the powder. Fluorescence was induced by TPA, using however a 600 nm excitation wavelength for better comparison of the results with previous experiments on stilbene.^33^ Spectra were measured with 1 nm resolution. Fluorescence spectra of bibenzyl powder at ambient pressure and up to 18 GPa are displayed in Figure 9. All of the spectra reported are smoothed for the sake of clarity. No bands ascribable to the monomer's emission are observed at shorter wavelengths other than the measured fluorescence peaked at ambient pressure at 355 nm and extending to 450 nm. Therefore, in the solid, as it was also for highly concentrated solutions (Figure 8a), the measured fluorescence is produced only from excimeric species. The spectral profile is in perfect agreement with those found in the literature,^2,22,54^ and the fluorescence spectrum at ambient pressure can be decomposed with 3 Voigt functions centered, respectively, at 354, 374, and 395 nm. A progression characterizes every measured spectrum with peaks separated by 1400–1500 cm^–1^, which is therefore assigned to the aromatic C=C stretching mode. As previously stated, the highest energy peak is cut off by the absorption band. As pressure is applied, this high-energy peak becomes visible, and at 1.2 GPa, it is centered at 348 nm. The energy gap with the next one at 361 nm (354 nm at ambient pressure) is ∼1000 cm^–1^, which suggests that reabsorption is still present, cutting the signal on the highest energy side and leading to an erroneous determination of the peak maximum. This peak is ascribed to the pure 0–0 electronic transition, as it was similarly observed for stilbene, for which such a band appears below a certain temperature^24,55^ or above a specific pressure value at a phase transition.^33^ The effects of the applied pressure are also seen on the structure of the emission profile, where the single components become much more defined with respect to the ambient pressure solid and bibenzyl solution; the same is observed on lowering the temperature.^56^

**Figure 9:**
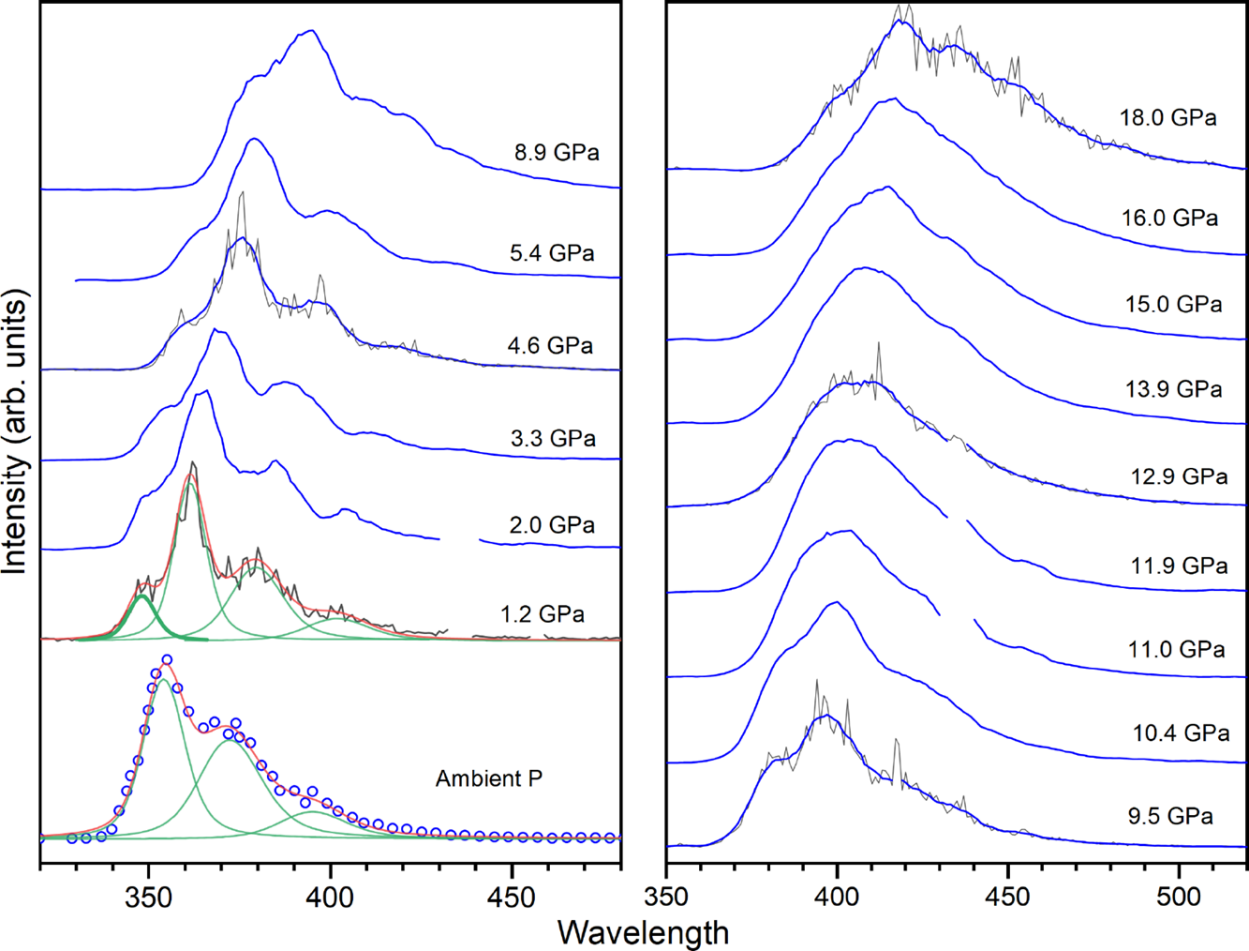
Fluorescence spectra measured for a bibenzyl powder sample up to 18 GPa. The spectra are normalized over the squared value of the pulse intensity at any given wavelength. The spectra are also normalized to 1 and smoothed for visual clarity (blue); the experimental data are shown in some spectra for comparison (gray). The ambient-pressure spectrum is measured on the sample outside of the DAC. This spectrum is deconvoluted using 3 Voigt functions (green), whereas a fourth Voigt function (thicker line) is employed to describe the short wavelength peak that appears with the application of pressure. The sum of the employed functions is also reported (red).

The fluorescence exhibits an evident red-shift with increasing pressure, which is a common occurrence in compression as intermolecular interactions in the excited state are enhanced by the density increase.^57,58^ In Figure 10, the pressure evolution of the peak maxima is shown; the emission shifts by about 4000 cm^–1^ from ambient pressure up to 13 GPa, after which a plateau is reached. The parameters for the linear regressions of the wavelength shift up to 13 GPa are reported in Table 1. This behavior has clear differences with what was observed for stilbene,^33^ whose emission shows a greater red-shift with pressure, shifting by 3800 cm^–1^ up to 9 GPa and by another 2900 cm^–1^, with a greater slope, up to 16 GPa. Differently also from stilbene,^33^ no new components appear during the compression and no drastic changes on the relative area of the bands are observed: only a small redistribution in favor of the highest energy peak that could be due to a reduction of reabsorption in compression. It is also interesting to follow the total area of the emission profile with increasing pressure, which is a direct probe of the efficiency of the fluorescence. As shown in Figure 10 the total area increases up to 12 GPa where a plateau is reached in close analogy with the peak shift (Table 1).

**Figure 10:**
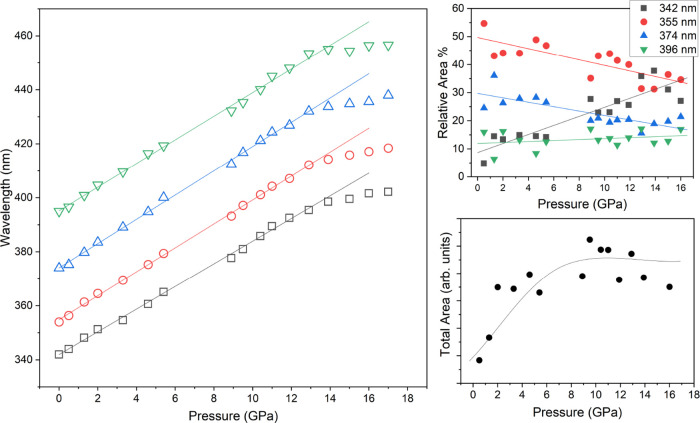
Left: Wavelength shift of the 4 Voigt functions used to decompose the emission profile as a function of pressure. Right: Variation of the relative area of the 4 Voigt functions employed (top) and evolution of the total absolute area of the emission profile in compression (bottom). The straight lines for the wavelength shift (left) and relative area variation (top right) are linear regressions of the associated data (Table 1), whereas the curve depicted in the total area evolution (bottom right) is drawn to serve as a guide to the eye.

**Table 1:** Slope and intercept of the linear regressions of the wavelength shift as a function of pressure reported in Figure 10 for each band^a^

λ = *aP* + *b*			
*a* (GPa^–1^)	*b* (nm)	*b* (cm^–1^)	difference *P* = 0 (cm^–1^)
4.20	341.98	29,241	
4.43	354.86	28,180	1061
4.49	374.06	26,733	1447
4.38	395.17	25,305	1428

a The value of intercept *b* is also reported in wavenumbers (cm^–1^) in addition to the energy separation in cm^–1^ for each band.

It is important to note that, although all of the fluorescence measurements were conducted on the same sample for the entirety of the compression, no signs of reaction and photodegradation were observed from the infrared spectra acquired before and after each irradiation (Figure S12).

## Discussion

SC-XRD and fluorescence measurements provide meaningful information about the structural and electronic properties of bibenzyl also in relation to the high-pressure reactivity. At the phase transition, detected at 13 GPa, there are elements of pseudosymmetry whose origin and effects can be rationalized on the basis of the intermolecular interactions, which, in the case of bibenzyl, greatly influence its fluorescence. Calculations from Zhang et al*.* ^22^ show that in the starting packing structure of the crystal, only weak intermolecular interactions are present. This is because the closest molecules in the crystalline packing are perpendicular to each other (T-shape), which does not favor excimer formation, while the molecules with favorable orientations are very far away. Therefore, a molecular motion driven by the transition dipole moment upon excitation has to occur to favor the formation of the detected excimeric species. This reorganization upon irradiation involves the same molecular movement as a function of pressure observed from the refined crystal structures, which essentially consists of a rotation of the molecules from perpendicular to a parallel arrangement, more favorable for excimer formation. Differently from stilbene, the frontier molecular orbitals present nodal plains at the ethylic moiety; thus, the excitation is entirely localized on the aromatic rings and does not get delocalized through the molecule. Because of this, the delocalization of the excitation onto suitably oriented neighbor molecules can be facilitated through intermolecular interactions, with the consequence of excimer formation.^59^ In the case of *trans*-stilbene instead, the excited state molecular orbitals develop throughout the entirety of the molecule, resulting in a strong delocalization of the excitation mainly through bonding π orbitals.^33^

In the crystal structure with the cell choice *P*2_1_/*n*, the interacting molecules are those along the *a*-axis direction, more specifically along the *ab* diagonal. From the calculated cell parameters (Figure 3), the cell is much more compressible along the *a* axis, moving the molecules along this direction closer to each other. In addition to this, the torsional angle of the phenyl rings greatly increases with pressure (Figure 6), reducing the perpendicularity between the molecules of interest. This also has direct consequences on the absolute measured intensity of the emission, which is a direct probe of the excimer formation efficiency. As reported in Figure 10, the emission intensity increases in compression up to ∼12 GPa, meaning that, with increasing pressure, the amount of emitting excimers increases as well. The evolution of the intensity coincides well with the structural evolution of the unit cell and of the molecules (Figures 3 and 6), where plateau values are reached above 10 GPa.

The pressure shift of the fluorescence provides important information on the structural evolution in compression, and in particular, it provides additional information to properly rationalize the high-pressure phase transition of bibenzyl. The observed red-shift with increasing pressure up to the phase transition is a common occurrence arising from a reduction of the energy gap between the ground state and the excited state due to the density increase that simultaneously stabilizes the excited state and destabilizes the ground state.^57,58^ After the phase transition around 13 GPa, neither the peak positions nor the intensity change anymore. This indicates that the excimer formation is not favored on further increasing the pressure, and it is derived from two factors. The first one is that the unit cell volume around the phase-transition pressure decreases at a slower rate, and therefore, the molecular conformation and intermolecular distances do not vary as much. Second, the formation of strong π–π interactions would lead to a stabilization of the ground state, which, with increasing pressure, would lower the energy uniformly with respect to the excimer states, thus counterbalancing the volume effects. Intermolecular π–π interactions would also be a leading cause for the appearance of pseudosymmetry after the phase transition,^60^ given that strong intermolecular interactions force molecular deformations, yielding a crystal structure with high *Z* and *Z*′ (*Z* = 6 and *Z*′=1.5 after the phase transition in this study).

Just below the phase-transition pressure, the closest centroid–centroid distances are equal to 4.06 and 4.33 Å, related to the aromatic ring in (1/2, 1/2, 1/2), with the closest rings at (1, 0, 0) and (0, 0, 0), respectively, with the relative orientation for both equal to 36.3° (Figure S14a). While the distances and angles are compatible with π–π interactions,^61,62^ the tilted relative orientation is less favorable with respect to a T-shaped or slipped-parallel configuration.^63^ After the phase transition, most distances and relative orientations between rings retain similar values (∼4.1 Å, ∼35°; Figure S14b). However, the interactions involving the C1′–C6′ ring of the distorted molecule with the closest rings are characterized by a significant decrease in their relative orientation angle. At 14 GPa, the smallest centroid–centroid distances for the C1′–C6′ ring are 4.05 Å with the closest symmetry equivalent rings and 4.55 Å with the closest symmetry nonequivalent ring (C1–C6), with a relative orientation for both of 30° (Figure S14c). At 37 GPa, the same distances become 3.77 and 4.38 Å, and the angle between them becomes 25.2° for both. Therefore, the changes observed in the crystalline structure mainly involve a rotation of the bibenzyl molecules changing the relative orientation leaning toward a slipped-parallel configuration, which is comparable in energy to a T-shaped configuration but much more stable than a tilted configuration,^61,63^ which occurs by applying pressure. At the phase transition, the accumulated stress is released with molecular distortions that favor π–π interactions between molecules, breaking the symmetry in the crystal and stabilizing the electronic ground state. These interactions evolve toward a parallel displaced arrangement, which, however, is not achieved at higher pressure according to the molecular evolution with increasing pressure (Figure 6). This would be the leading cause for the reduced reactivity of bibenzyl as opposed to stilbene and azobenzene, where high-pressure polymerization occurs along a specific direction where the molecules are parallel-displaced, thus favoring π-stacking interactions.^45^

Lastly, the Raman-active lattice phonon modes measured in a powder sample, reported in ref 21, are in accordance with the structural changes occurring at the phase transition. In the low-pressure phase, 6 Raman phonon modes are expected and measured according to the group theory (*Z* = 2, *C*_*i*_ point group, *C*_*i*_ site symmetry, *C*_2*h*_ space group, 6 Raman, and 3 IR phonon modes expected). After the phase transition, 18 Raman phonon modes are measured and are also expected from the group theory if we consider the 6 Raman phonon modes from the molecules that retain the *C*_*i*_ point group symmetry and are still in the *C*_*i*_ site, in addition to the distorted molecules with *Z* = 4 with *C*_1_ symmetry in *C*_1_ sites that should produce 12 Raman and 9 IR active phonon modes.

## Conclusions

Bibenzyl (1,2-diphenylethane) was compressed in quasi-hydrostatic conditions with helium as a PTM, and its structural evolution was studied by means of SC-XRD and TPA-induced fluorescence. Bibenzyl structures are refined up to 37 GPa, showing a much higher compressibility along the *a*-axis direction and a phase transition occurring at 13 GPa. At the phase transition, molecular distortions occur and the point group symmetry of specific molecules is lowered, leading to pseudosymmetry in the unit cell, which is refined with the same space group as the low-pressure phase, but with triple its *a* cell parameter and its *Z* value, passing from 2 to 6. The molecular distortions that occur can be rationalized from the bibenzyl crystal structures and fluorescence. The emission of bibenzyl originates from excimer species, and the evolution of the fluorescence in compression mirrors the changes in intermolecular interactions. The fluorescence shift and intensity greatly increase during compression, which is a sign of more efficient excimer formation and stabilization with increasing pressure. After the phase transition, despite a non-negligible volume reduction of the unit cell in compression, the emission shift and intensity stop changing. This is ascribed to the strengthening of π–π intermolecular interactions, which drive the molecular distortions in a specific way. Ultimately, it is the added mobility of the molecule granted by the ethane moiety that permits this behavior at the phase transition, as opposed to a rearrangement of the molecules in the unit cell with major changes in the unit cell parameters, as it is for more rigid molecules such as azobenzene.^46^ However, the formation of π–π interactions at the phase transition mainly involves pairs of molecules and does not extend in the crystal structure, which is therefore the main reason for the reduced reactivity with respect to other pseudostilbenes. This study provides a full description of the bibenzyl crystal evolution as a function of pressure, with a complete rationalization of the structural changes and of the emission properties in compression, laying the groundwork for the synthesis and characterization of carbon nanothreads with tunable properties from mixed crystals of pseudostilbene compounds.

## Methods

### SC-XRD Measurement

For the SC-XRD measurements, bibenzyl single crystals were recrystallized from the slow evaporation of ethanol solutions. For the synchrotron measurements, two bibenzyl crystals are loaded in a 150 μm rhenium gasket mounted on a DAC equipped with 350 μm culet diamonds and a 60° aperture. Due to the high volatility of bibenzyl, the two crystals were loaded inside the DAC on site. A small ruby chip is loaded together with the crystals to measure the pressure inside the sample chamber during the compression by using the fluorescence shift of the ruby.^64^ Helium is then gas-loaded inside the sample chamber as a PTM. High-pressure SC-XRD measurements were performed at the ID15B beamline^65^ at the ESRF synchrotron with 0.41 Å radiation and a 4 μm beam diameter. A large area EIGER2 × 9 M CdTe (340 × 370 mm) flat panel detector was used, and the detector distance was 180 mm, calibrated using a silicon standard. Before the experiments, a vanadinite single crystal was used as a standard for the calibration of the detector parameters for data reduction. The diffraction patterns were collected in compression up to 37 GPa with an increasing pressure step ranging from 0.5 to 2 GPa. After the sample was compressed to the highest reached pressure, it was left for one full day at that pressure and then decompressed, collecting measurements along the decompression path. The decompressed sample could not be recovered outside of the DAC. The patterns were measured with a sample rotation of 60° (− 30°, + 30°) with a 0.5° step. The acquisition time was 2 s for the measurements up to 1 GPa; after that, the acquisition time was increased to 5 s. CrysAlis^*PRO*^ software^66^ was used for indexing reflections and for data reduction. The crystal structures were refined with the SHELXL program^67^ through Olex2 software.^68^ The completeness achieved was ∼20% for all refined structures with slight deviations. The hydrogen atoms were refined, riding in their idealized position, whereas all of the other carbon atoms were refined in anisotropic approximation. No geometrical constraint and no restraint on the ADPs were set during the refinements at the cost of a worse data/parameter ratio, which has been deemed to be sufficient even at the highest pressure values. Only the structure at 8.6 GPa was refined with the *AFIX* geometrical constraint due to an anomalous data set. Calculations and measurements on the refined structures were performed with Mercury software^50^ and Platon program.^19^

For in-house single-crystal measurements, a Bruker D8 Venture equipped with the IμS 3.0 X-ray source with Mo radiation and the 100 × 140 mm^2^ detector (PHOTON II) was used. A bibenzyl single crystal was mounted on a transparent microloop to achieve near completeness in data collection, and the measurement was conducted under a cold nitrogen flux (173 K). The measurements were carried out at the *Réseau des Rayons X et Gamma (RRXG)* part of the *Plateforme d’Analyse et de Caractérisation (PAC)* in the *Institut Charles Gerhardt Montpellier (ICGM)*. The ambient-pressure structure from this measurement was obtained using *olex2.refine* as the refinement software and without imposing any constraint/restraint. Nonspherical form factors were employed and calculated with the *NoSphera2* function^69,70^ using *Orca* ^71^ for the calculations.

Full structural information has been deposited with the Cambridge Crystallographic Data Centre. CCDC-2420532, 2466021 – 2466043, 2473990, and 2516032 – 2516038.

### TPA-Induced Fluorescence Setup

TPA-induced fluorescence spectroscopy was employed to monitor the pressure evolution of the electronic properties of bibenzyl. TPA is a third-order nonlinear process and is proportional to the squared intensity of the incident light; thus, it is strongly dependent on the energy density of the beam that is the highest at its focus. Therefore, TPA is induced only in the focus proximity opposed to OPA, where the spatial distinction is not critical. This allows the selection of fluorescence from the sample in the DAC and background contributions that are minimized. In addition, the low cross section of the process that produces a lower number of excited species in the sample compared to OPA permits a better control over photoinduced reactions that may degrade the sample at high pressure.^31,32,72–74^ Two-photon-induced fluorescence was measured on a single bibenzyl powder sample, loaded in a 150 μm gasket in a DAC and compressed up to 18 GPa. The 355 nm third harmonic of a Nd:YAG pulsed laser (PL2143A from EKSPLA) was used to pump an optical parametric generator/amplifier (OPG/OPA). The pulses exit the Nd:YAG pump laser with vertical polarization, a duration of 80 ps, beam waist around 8 mm, energy up to 20 mJ, and a 10 Hz repetition rate. In the OPG, the pump pulses shine on an LBO crystal (LiB_3_O_5_) generating through nonlinear optical processes, an *idler* and a *signal* radiation. While the idler portion gets discarded, the signal radiation is used, and its wavelength can be varied from 420 to 680 nm by changing the orientation of the LBO crystal. The OPG output of 30 ps pulses and ∼0.5 mJ energy is then sent to the sample and filtered, reaching a final energy of tens of microjoules by means of a pinhole and neutral optical density filters. Before reaching the sample, 3% of the excitation radiation was reflected by a beam sampler onto a silicon photodiode (S1722-02 by Hamamatsu) used for the fluorescence signal normalization. The beam is focused onto the sample in the DAC by means of an achromatic doublet with a focal length of 100 mm through a pierced parabolic mirror. The parabolic mirror with a 50 mm focal length collects the emission from the sample in a backward geometry, sending it to a 300 grooves/mm holographic grating single-stage monochromator (Oriel CS260 1/4m) with a 1 nm resolution. At the entrance of the monochromator, a 575 nm short-pass filter is placed to filter out the residues of the excitation wavelength. The signal is detected by a photomultiplier and read by an oscilloscope.

The ruby chip for measuring the effective pressure inside the cell is not used to avoid interference in the fluorescence measurements. The pressure was determined by the peak frequency of the two IR bands, at ambient pressure, at 1025 and 1060 cm^–1^.^21^ These two bands originate, respectively, from out-of-plane and in-plane C–H bending. The pressure shift of these bands was previously determined by measuring the FTIR spectra using the ruby fluorescence method for pressure calibration (Figure S13). Infrared absorption spectra were measured before and after every fluorescence measurement with the additional purpose of determining sample degradation under pulsed irradiation.

Type IIac diamonds from Almax-Easylab were employed for the fluorescence measurements, which present the UV absorption edge around 230 nm and ensure transparency in the UV–visible region of interest (∼300 to 550 nm).

## Supplementary Material



## Notes

Bibenzyl unit cell and labeling (Figure S1), ethane bond length as a function of pressure (Figure S2), image of the loaded sample in DAC for synchrotron SC-XRD (Figure S3), comparison between powder data from ref 21 and single-crystal data (Figure S4), high-pressure phase unit cell and molecule labeling (Figure S5), ADPs *U*_*ii*_ diagonal terms as a function of pressure (Figure S6), *U*_eq_s of all carbon atoms as a function of pressure (Figure S7), 2D sum images of the SC-XRD patterns (Figure S8), azimuthally integrated XRD patterns at phase-transition and high pressure (Figure S9), absorption spectra of bibenzyl and stilbene solutions (Figure S10), absorption spectra of bibenzyl solutions with increasing water fractions (Figure S11), IR spectra before and after the TPA-induced experiments (Figure S12), selection of vibrational mode shifts for pressure calibration (Figure S13), visualization of the most relevant intermolecular interactions (Figure S14); relevant collection and refinement parameters for in-house and synchrotron XRD measurements from ambient pressure up to 37 GPa (Tables S1 and S2), and the same parameters for the structures at 14.0, 14.8, and 15.8 GPa refined without satellite reflections (Table S3) (PDF)
