# Supplementary material for: High-Pressure Structural and Electronic Properties of Bibenzyl (1,2-Diphenylethane) from Synchrotron SC-XRD and Two-Photon-Induced Fluorescence
Source: Cryst Growth Des. 2026 Jan 8;26(2):970–84. doi: 10.1021/acs.cgd.5c01569 (PMC12833920; doi:10.1021/acs.cgd.5c01569)
Supplement: Supplementary file 1 [file cg5c01569_si_001.pdf]

Supporting Information: High-pressure structural and electronic properties of bibenzyl (1,2-diphenylethane) from synchrotron SC-XRD and two-photon induced fluorescence

Milo Agati,[*,†,‡] Sebastiano Romi,[†,‡] Samuele Fanetti,[†,¶] Gaston Garbarino,[§] Julien Haines,[‖] and Roberto Bini[†,‡,⊥]

†*LENS, European Laboratory for Non-linear Spectroscopy, Via N. Carrara 1, I-50019 Sesto Fiorentino, Firenze, Italy*

‡*Dipartimento di Chimica "Ugo Schiff", Università di Firenze, Via della Lastruccia 3, I-50019 Sesto Fiorentino, Italy*

¶*ICCOM-CNR, Istituto di Chimica dei Composti OrganoMetallici, Via Madonna del Piano 10, I-50019 Sesto Fiorentino, Firenze, Italy*

§*European Synchrotron Radiation Facility, ESRF, 71 Avenue des Martyrs, CS40220, 38043 Cedex 9 Grenoble, France*

‖*ICGM, UMR5253 , CNRS, Université de Montpellier, ENSCM, 1919 route de Mende - 34293 Montpellier cedex 5*

⊥*INO-CNR, Istituto Nazionale di Ottica, Via N. Carrara 1, I-50019 Sesto Fiorentino, Firenze, Italy*

E-mail: agati@lens.unifi.it

Supplementary information

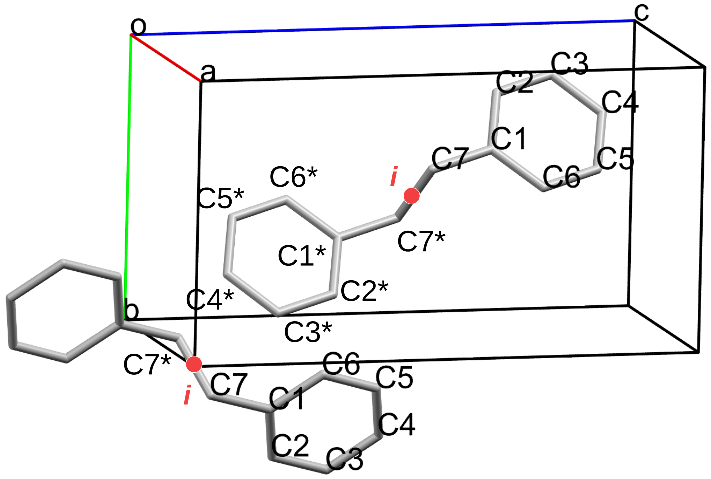

Figure S1: Bibenzyl unit cell at ambient pressure. The labeling used for this work is shown in the image. One half of the molecule is numbered C1 through C7 while the other C1* through C7* as it is symmetry generated by the inversion center on the C7C7* ethane group.

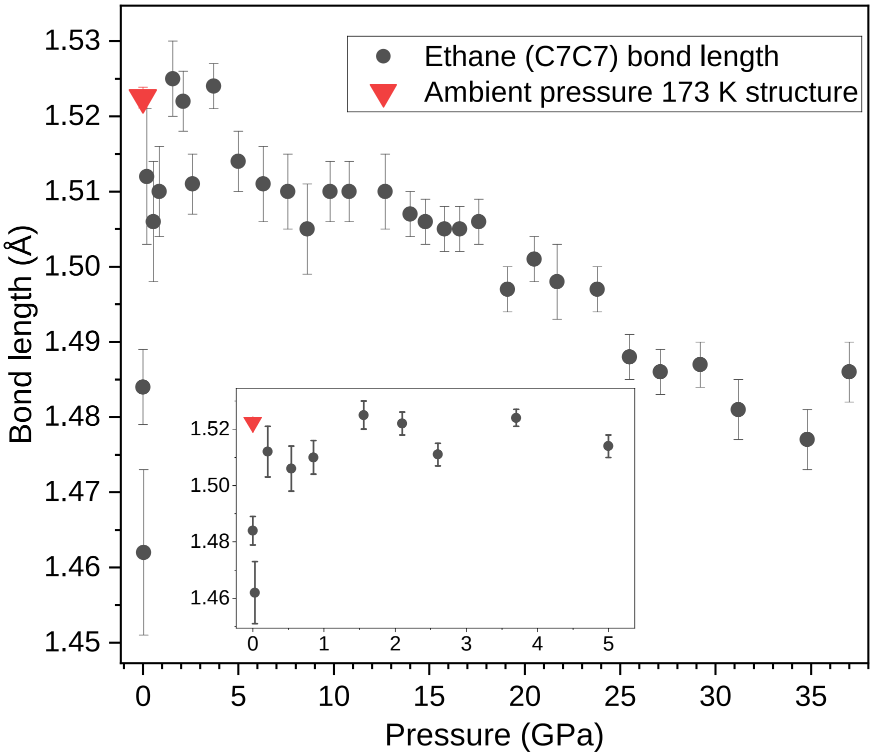

Figure S2: Ethane bond (C7−C7*) length as a function of pressure (black dots). The bond at ambient pressure and temperature has an unusual short length likely originating from the dynamic disorder. The oscillations of the bond length values with increasing pressure occur from the competing effects from the reduction of the U_{eq} that enhances bond length precision, and from the actual reduction of the distances with the increasing pressure. The ethane bond length measured at ambient pressure and 173 K is shown with a downward pointing red triangle symbol. The error for this value is within the symbol size.

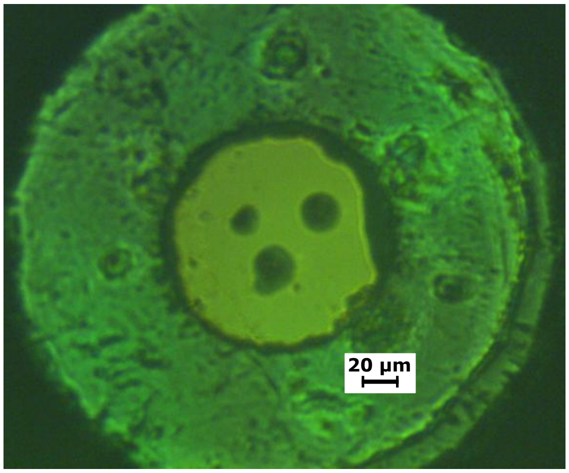

Figure S3: DAC's sample chamber after gas-loading with helium (0.03 GPa). Two bibenzyl single crystals are loaded (top-left and bottom) together with a big ruby chip (right). The smaller crystal (top-left) broke around 2 GPa while the bigger one (bottom) was compressed up to 37 GPa and data were successfully collected.

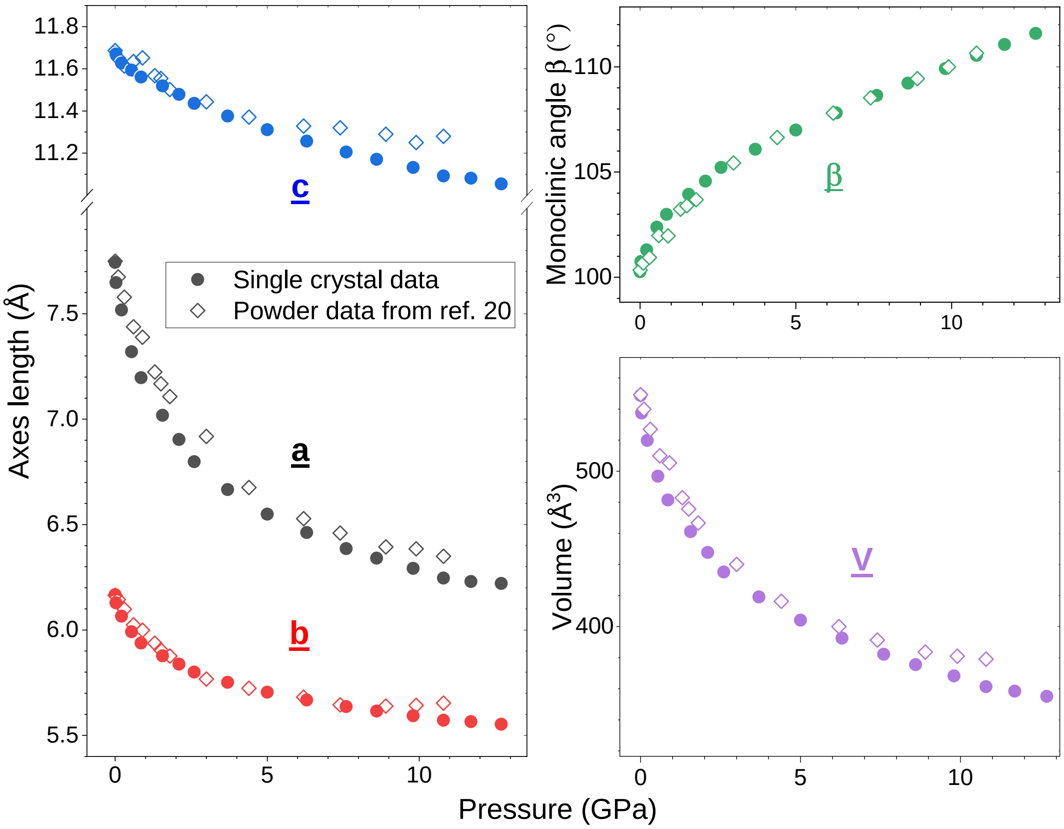

Figure S4: Comparison between data obtained from the single crystal XRD measurements presented in the main text (filled circles) and data from powder XRD presented in ref.21 of the main text (empty lozenges). Slight discrepancies between the two datasets arise from employing a non-hydrostatic compression, in the case of the powder experiment, which leads to worse quality of the patterns at higher pressure values and to a systematic error to the pressure determination of ±0.2 GPa ca. due to the measurement of a broader fluorescence from a much more stressed ruby chip.

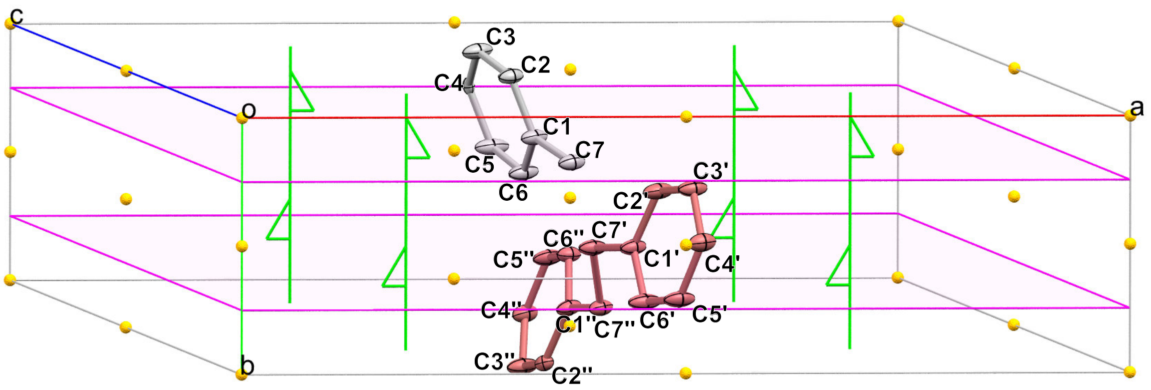

Figure S5: New asymmetric unit after the phase transition at 13 GPa with the relative labeling. The asymmetric unit becomes composed by two non symmetry-equivalent molecules with one of them positioned on inversion centers at the Wyckoff position 2a (grey, labelled C1 through C7) and the other in a general Wyckoff position 4e (red, labelled C1' through C7' for one half of the molecule and C1" through C7" for the other half). The thermal ellipsoids are shown in the figure with the average equivalent isotropic displacement parameters being ∼0.2Å^2 (see Figure S7). All the symmetry elements in the unit cell are shown: the inversion centers in yellow, screw axes in green and glide planes in magenta.

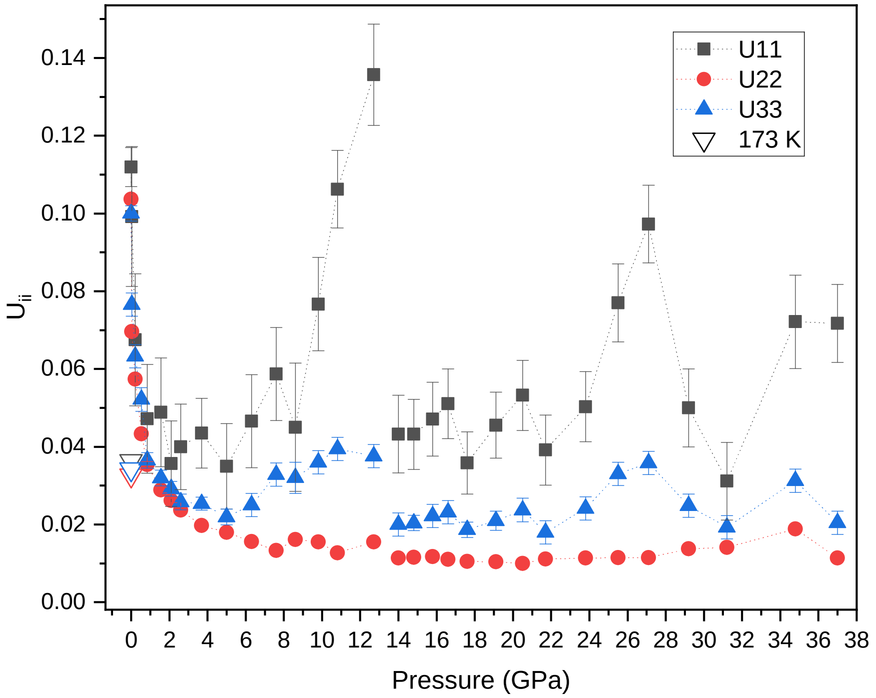

Figure S6: Diagonal terms of the anisotropic displacement parameters (ADP) tensor for the same molecule before and after the phase transition (C1-C7). U_{11}, U_{22}, U_{33} are the components pointing toward the a, b, c axes respectively. As pressure increases, the U_{11} component becomes greater than the other two and is in agreement with a torsional movement and molecular vibrations mainly occurring in the direction of the a axis. After the phase transition the value of U_{11} is still larger than the other terms but much more contained. Higher values above 24 GPa are due to a quality worsening of the data and also to the decreased hydrostaticity of helium at higher pressure values. The off-diagonal terms are not reported for clarity reasons, and are smaller or comparable to the U_{22} and U_{33} terms. Downward pointing triangle symbols characterize the diagonal terms of the ADP tensor for the structure refined at ambient pressure and 173 K. Error bars if not shown are within the symbol size.

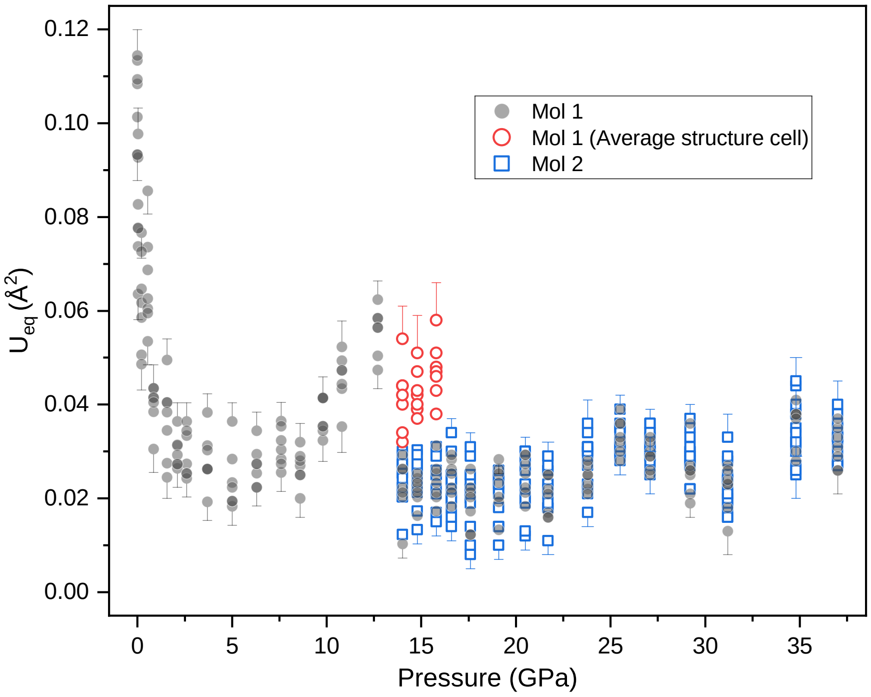

Figure S7: Equivalent isotropic displacement parameters (U_{eq}) as a function of pressure. The semi-transparent gray points are related to the carbon atoms of the molecules at Wyckoff position 2a (0, 0, 0 and 1/2, 1/2, 1/2) both in the low- and high-pressure phase. The empty red circle refer to the same molecule but for the average structure refined without satellites reflections. The empty blue square are instead related to the symmetry non-equivalent molecule that is refined in the high-pressure phase. The values of the U_{eq} for the two symmetry non-equivalent molecules in the high-pressure structure are very similar while the U_{eq} values of the carbons in the refined average cell (without satellites reflection) are much higher. This is because this structure is refined in the same space group but has to account for molecular distortions occurring at the phase transition. Only the top and bottom error bar for each pressure point is shown for vision clarity and the errors are extremely similar between carbon atoms of the same refined structure

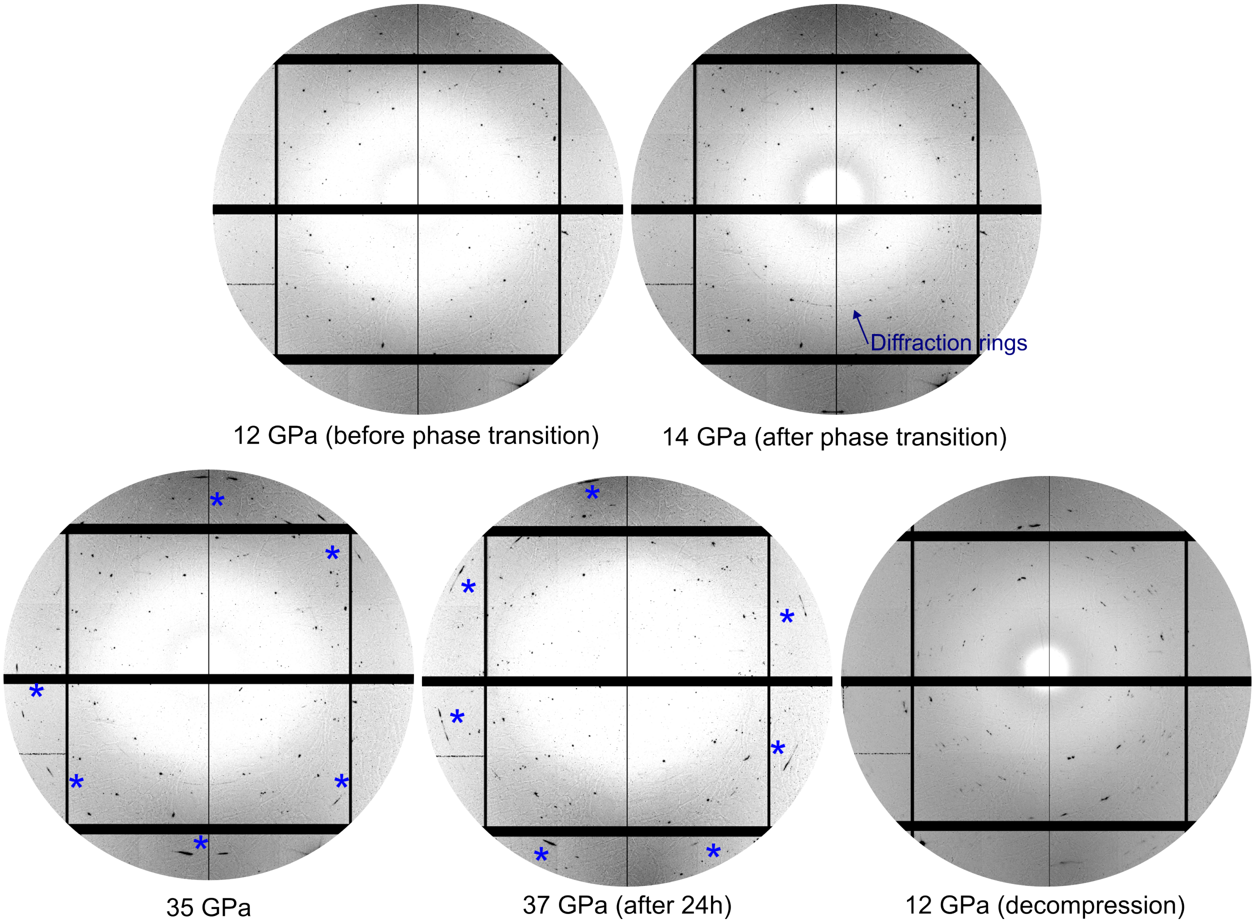

Figure S8: 2D sum image of the x-ray diffraction patterns acquired with a rotation of 60° (-30°, +30°) and a 0.5° step. Weak diffraction rings can be observed after the phase transition and, at the highest pressures, a ring with bright spots in an octahedral geometry is observed. This one becomes more distinct after keeping the sample for 24 hours at the highest pressure. Crossing back the phase transition in decompression (12 GPa) all of these observed features disappear.

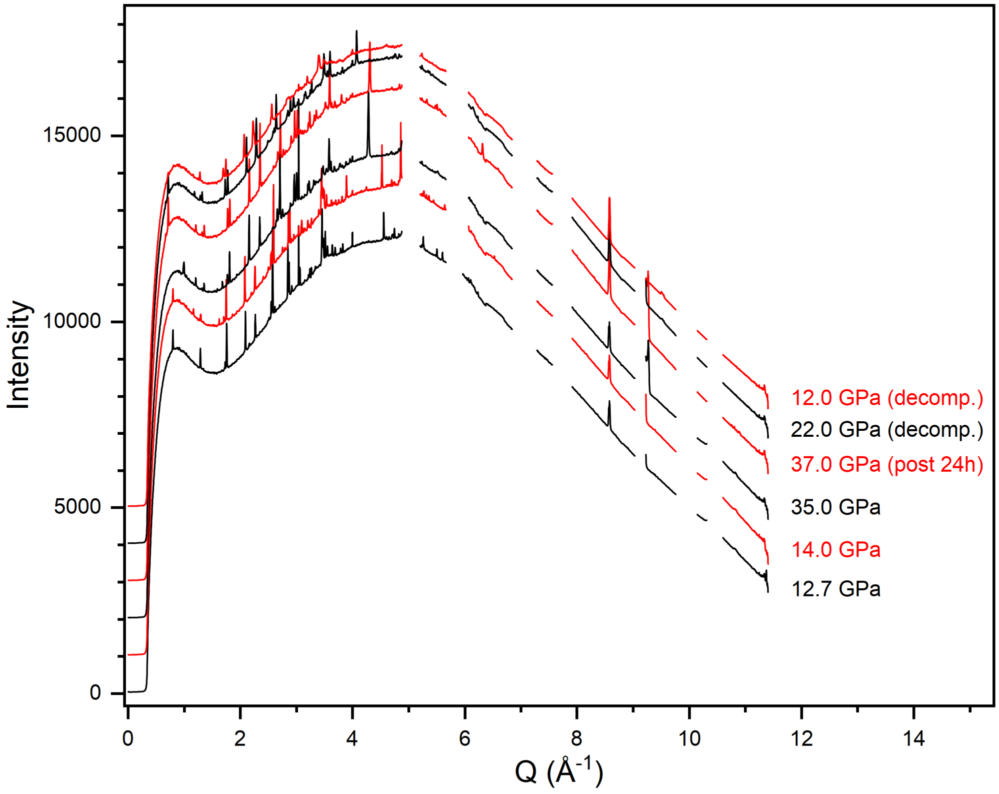

Figure S9: Azimuthally integrated X-ray diffraction patterns at specific pressure values without removing the background. The diffractograms are offset by a set amount between them (1000 units). The background oscillates between pressure points making it difficult to determine the formation of amorphous material. Nonetheless, specific pressure points are checked. Below (12.7 GPa) and above (14 GPa) the phase transition pressure no changes are observed, nor increasing pressure up to 35 GPa. After 24 hours (37 GPa) a small increase in the background component is observed, however it is unclear whether this arises from the formation of an amorphous material. In decompression, no clear signs of an amorphous material are observed crossing the phase transition pressure again between 22 GPa and 12 GPa.

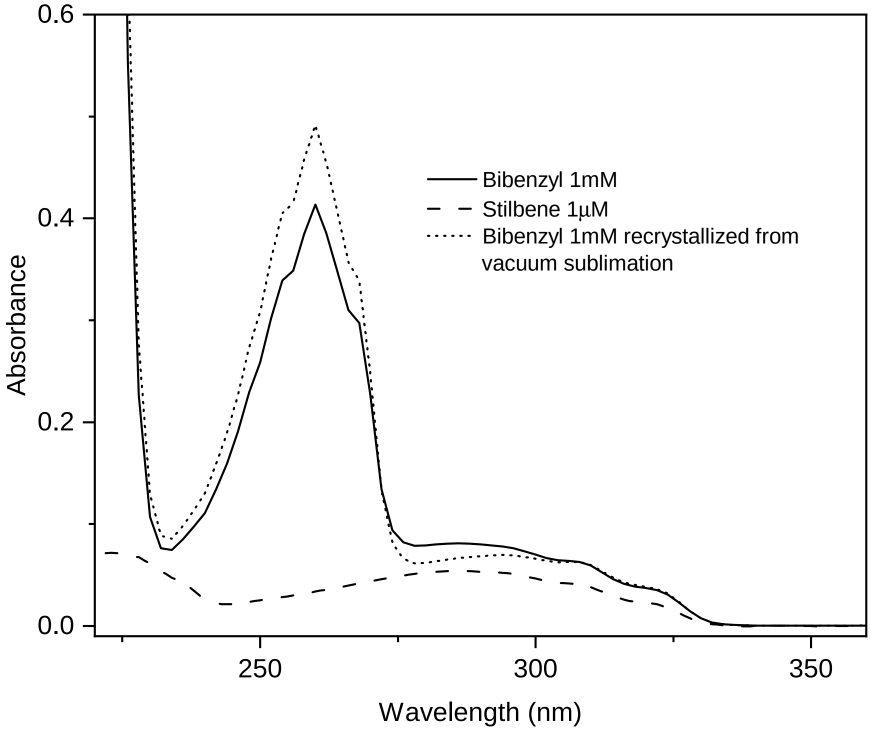

Figure S10: Absorption spectra of solutions in ethanol of bibenzyl (1 mM, full line) and stilbene (1 µM, dashed line). The absorption band in bibenzyl around 300 nm is ascribed to small presence of stilbene impurities which, comparing the two spectra, are less than 0.5%. Crystalline bibenzyl purchased from Sigma-Aldrich Lot #STBH6120 is certified having 99.8% purity, however contamination over time may have occurred. The absorption spectrum of a 1 mM solution in ethanol of purified bibenzyl is also reported (dotted line). Bibenzyl has been purified by recrystallization onto a cold finger from vacuum sublimation. Discrepancies between spectra may be ascribed to errors in the concentration values.

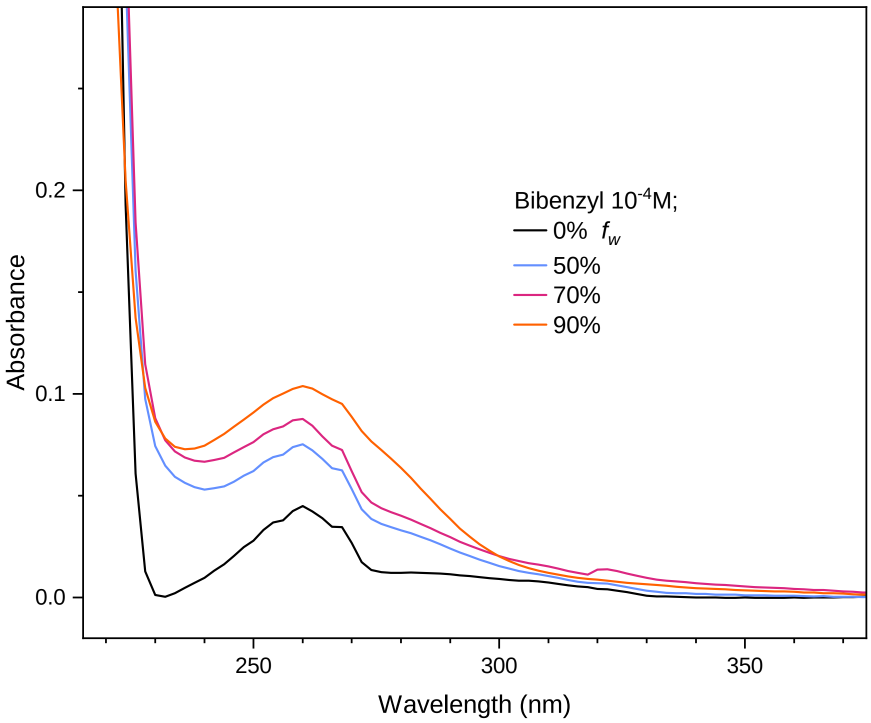

Figure S11: Absorption spectra of the 0.1 mM bibenzyl solutions in different ethanol/water fractions. Only the 0% f_w has the blank ethanol component removed. Slight differences in the background are ascribed to the different ethanol/water fractions while at 90% f_w only a slight broadening is observed.

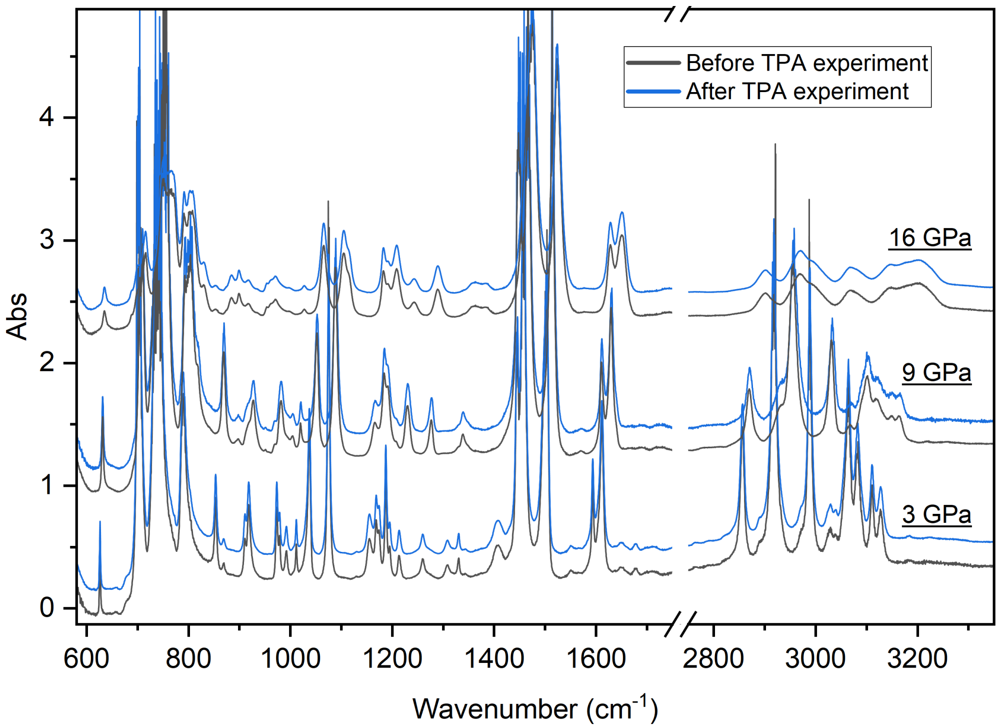

Figure S12: Infrared spectra before (black) and after (blue) the TPA-induced fluorescence measurement. Only a few spectra covering the low-, medium- and high-pressure regime are presented for visual clarity. The spectra are spaced between pressure points and between TPA induced fluorescence measurements. No changes are observed indicating that no reactivity is induced by the irradiation.

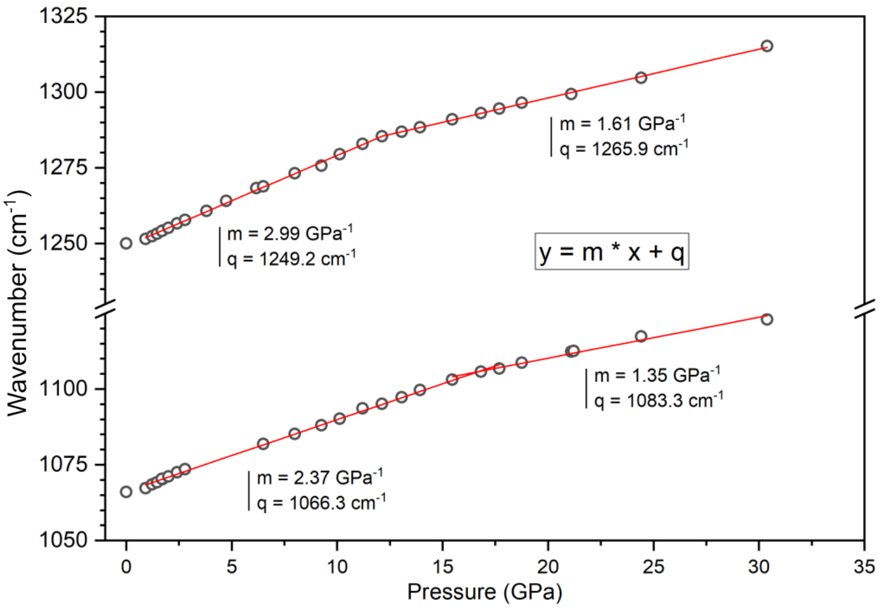

Figure S13: Wavenumbers of a selection of vibrational modes obtained by deconvolution with Voigt function. These modes are related to in-plane C-H bending (1060 cm^{-1}) and C-Ph stretching (1250 cm^{-1}) modes. The linear regressions are shown with red lines and the relative related paramters are reported in the insets. More bands are used to obtain a more accurate estimate of the pressure inside the sample chamber.

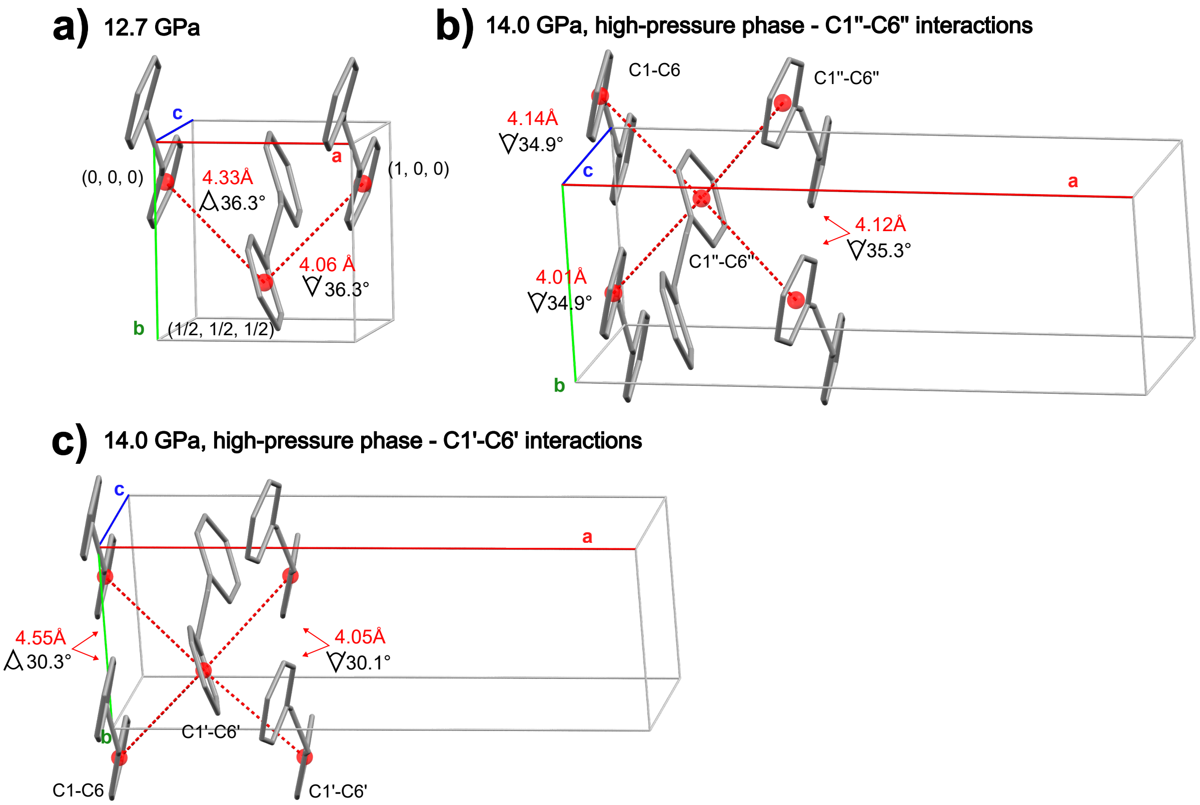

Figure S14: Visualization of the most relevant π-π interactions before and after the phase transition. a) Interactions in the low pressure phase at 12.7 GPa: although the distances are compatible with π-π interactions, the tilted configuration is not as favorable as slipped parallel or T-shaped. b) Interactions in the high-pressure phase involving the C1"-C6" ring: after the phase transition not many changes regarding this ring's interactions are observed. c) Interactions in the high-pressure phase of the C1'-C6' ring: although the distances between ring centroids do not present significant changes, the relative orientation between rings has an abrupt reduction of 6° degrees, therefore moving toward a more stable slipped parallel configuration.

Table S1: Table with all the relevant collection and refinement data for the bibenzyl crystal at ambient pressure on a micro-loop mount. The data were collected at low temperature (173 K) under a stream of cold nitrogen gas.

Pressure	Ambient (loop mount)	ρ_{calc} (g cm^{-3})	1.141
CCDC number	2473990	Z, Z'	2 / 0.5
Temperature	173 K	Reflections measured, independent, observed [I > 2σ(I)]	19496 / 2726 / 1815
Radiation (Å)	0.71073, in-house Mo	θ range (°)	3.56 / 38.20
a (Å)	7.5072(5)	Data completeness θ_{full} = 14.237 / θ_{max}	0.9738 / 0.9339
b (Å)	6.1804(3)	R_{int}	0.0503
c (Å)	11.6373(6)	R1 [F^2 > 2σ(F^2)], wR2 (F^2), GooF	0.0491 / 0.1239 / 1.0496
β (°)	100.781(5)	Parameters / restraints	64 / 0
V (Å^3)	530.41(5)	ρ_{max} / ρ_{min} (e Å^{-3})	0.2391 / -0.2373

Table S2: Table with collection and refinement data. Measurements done at ambient temperature (298 K)

Pressure (GPa)	Ambient	0.03	0.2	0.5	0.8	1.5
CCDC number	2466021	2420532	2466022	2466034	2466043	2466033
Radiation (Å)	0.4099, synchrotron					
a (Å)	7.744(2)	7.648(11)	7.517(8)	7.325(8)	7.197(8)	7.018(5)
b (Å)	6.1672(3)	6.1283(5)	6.0653(3)	5.9908(3)	5.9382(3)	5.8772(2)
c (Å)	11.6766(16)	11.670(4)	11.629(2)	11.597(2)	11.561(3)	11.5190(19)
β (°)	100.265(19)	100.75(8)	101.30(6)	102.32(6)	102.99(6)	103.94(4)
V (Å^3)	548.76(17)	537.4(8)	519.9(5)	497.2(5)	481.5(6)	461.1(4)
ρ_{calc} (g cm^{-3})	1.103	1.126	1.164	1.217	1.257	1.313
Z, Z'	2 / 0.5					
Reflections measured,<br>independent,<br>observed [I > 2σ(I)]	1157<br>685<br>290	1057<br>525<br>298	1047<br>514<br>346	976<br>493<br>331	929<br>480<br>380	917<br>452<br>380
θ range (°)	2.162 / 20.912	2.173 / 20.720	2.193 / 20.784	2.218 / 20.333	2.236 / 20.340	2.258 / 20.559
Data completeness<br>θ_{full} = 14.237 / θ_{max}	0.394 / 0.226	0.308 / 0.182	0.311 / 0.182	0.307 / 0.195	0.312 / 0.195	0.310 / 0.186
R_{int}	0.0239	0.0445	0.053	0.0478	0.0338	0.0277
R1 [F^2 > 2σ(F^2)],<br>wR2 (F^2),<br>GooF	0.0509<br>0.1421<br>0.911	0.0632<br>0.1855<br>0.959	0.0665<br>0.1967<br>1.049	0.0625<br>0.1898<br>1.003	0.0639<br>0.1929<br>1.098	0.0585<br>0.1753<br>1.085
Parameters / restraints	64 / 0	64 / 0	64 / 0	64 / 0	64 / 0	64 / 0
ρ_{max} / ρ_{min} (e Å^{-3})	0.065 / -0.066	0.079 / -0.064	0.083 / -0.097	0.107 / -0.108	0.126 / -0.098	0.153 / -0.131

Table S2 continuation.

Pressure (GPa)	2.1	2.6	3.7	5.0	6.3
CCDC number	2466029	2466027	2466035	2466031	2466038
Radiation (Å)	0.4099, synchrotron				
a (Å)	6.903(5)	6.798(4)	6.666(5)	6.549(3)	6.462(2)
b (Å)	5.8377(2)	5.80000(10)	5.75170(10)	5.70490(10)	5.66810(10)
c (Å)	11.479(2)	11.4362(18)	11.376(2)	11.311(3)	11.257(3)
β (°)	104.57(4)	105.22(4)	106.08(5)	106.99(3)	107.81(3)
V (Å^3)	447.7(3)	435.1(3)	419.1(3)	404.2(2)	392.56(17)
ρ_{calc} (g cm^{-3})	1.352	1.391	1.444	1.498	1.542
Z, Z'	2 / 0.5				
Reflections measured,<br>independent,<br>observed [I > 2σ(I)]	823<br>423<br>381	816<br>422<br>377	769<br>394<br>357	754<br>394<br>357	731<br>384<br>358
θ range (°)	2.273 / 20.672	2.288 / 20.812	2.308 / 20.996	2.328 / 21.177	2.344 / 20.508
Data completeness<br>θ_{full} = 14.237 / θ_{max}	0.289 / 0.177	0.300 / 0.178	0.282 / 0.168	0.305 / 0.171	0.311 / 0.187
R_{int}	0.0162	0.0169	0.0124	0.0102	0.0105
R1 [F^2 > 2σ(F^2)],<br>wR2 (F^2),<br>GooF	0.0483<br>0.1478<br>1.094	0.0545<br>0.1537<br>1.071	0.0482<br>0.1322<br>1.052	0.0557<br>0.1641<br>1.101	0.0601<br>0.1763<br>1.124
Parameters / restraints	64 / 0	64 / 0	64 / 0	64 / 0	64 / 0
ρ_{max} / ρ_{min} (e Å^{-3})	0.164 / -0.119	0.193 / -0.126	0.189 / -0.126	0.251 / -0.144	0.346 / -0.159

Table S2 continuation.

Pressure (GPa)	7.6	8.6	9.8	10.8	12.7
CCDC number	2466039	2466025	2466028	2466023	2466037
Radiation (Å)	0.4099, synchrotron				
a (Å)	6.386(2)	6.341(2)	6.292(2)	6.246(2)	6.221(3)
b (Å)	5.63620(10)	5.61530(10)	5.59310(10)	5.57130(10)	5.5530(2)
c (Å)	11.205(3)	11.171(3)	11.132(3)	11.092(3)	11.054(4)
β (°)	108.64(3)	109.22(3)	109.91(3)	110.54(3)	111.58(4)
V (Å^3)	382.17(16)	375.57(17)	368.33(17)	361.44(18)	355.1(2)
ρ_{calc} (g cm^{-3})	1.584	1.612	1.643	1.675	1.704
Z, Z'	2, 0.5				
Reflections measured,<br>independent,<br>observed [I > 2σ(I)]	714<br>372<br>343	704<br>360<br>331	643<br>346<br>316	627<br>331<br>304	610<br>326<br>291
θ range (°)	2.359 / 20.574	2.370 / 20.615	2.381 / 20.663	2.392 / 20.087	2.404 / 20.750
Data completeness<br>θ_{full} = 14.237 / θ_{max}	0.306 / 0.185	0.301 / 0.181	0.289 / 0.176	0.282 / 0.185	0.275 / 0.169
R_{int}	0.0138	0.0126	0.0252	0.0211	0.0227
R1 [F^2 > 2σ(F^2)],<br>wR2 (F^2),<br>GooF	0.0565<br>0.1730<br>1.172	0.0757<br>0.2110<br>1.102	0.0609<br>0.1660<br>1.028	0.0552<br>0.1475<br>1.076	0.0703<br>0.1801<br>1.089
Parameters / restraints	64 / 0	52 / AFIX	64 / 0	64 / 0	64 / 0
ρ_{max} / ρ_{min} (e Å^{-3})	0.244 / -0.161	0.230 / -0.207	0.178 / -0.157	0.248 / -0.173	0.186 / -0.181

Table S2 continuation.

Pressure (GPa)	14.0	14.8	15.8	16.6	17.6
CCDC number	2466032	2466042	2466041	2466024	2466040
Radiation (Å)	0.4099, synchrotron				
a (Å)	18.884(8)	18.821(7)	18.750(7)	18.687(6)	18.616(7)
b (Å)	5.56600(10)	5.55420(10)	5.54400(10)	5.53220(10)	5.52070(10)
c (Å)	10.9566(3)	10.9256(3)	10.9004(2)	10.8754(2)	10.8518(2)
β (°)	115.243(12)	115.597(10)	115.897(11)	116.089(9)	116.294(9)
V (Å^3)	1041.7(4)	1030.0(4)	1019.3(4)	1009.7(3)	999.9(4)
ρ_{calc} (g cm^{-3})	1.743	1.763	1.781	1.798	1.816
Z, Z'	6, 1.5				
Reflections measured,<br>independent,<br>observed [I > 2σ(I)]	2086<br>1053<br>894	2032<br>1034<br>880	1944<br>1016<br>841	1960<br>1003<br>848	1897<br>967<br>817
θ range (°)	2.175 / 19.981	2.179 / 20.026	2.183 / 20.064	2.187 / 20.105	2.191 / 20.145
Data completeness<br>θ_{full} = 14.237 / θ_{max}	0.320 / 0.208	0.315 / 0.205	0.315 / 0.203	0.314 / 0.201	0.300 / 0.194
R_{int}	0.0209	0.0195	0.0184	0.0253	0.0214
R1 [F^2 > 2σ(F^2)],<br>wR2 (F^2),<br>GooF	0.0536<br>0.1611<br>1.032	0.0520<br>0.1411<br>1.009	0.0501<br>0.1385<br>1.021	0.0500<br>0.1364<br>1.022	0.0462<br>0.1207<br>1.014
Parameters / restraints	190 / 0	190 / 0	190 / 0	190 / 0	190 / 0
ρ_{max} / ρ_{min} (e Å^{-3})	0.251 / -0.160	0.228 / -0.176	0.203 / -0.143	0.227 / -0.139	0.205 / -0.128

Table S2 continuation.

Pressure (GPa)	19.1	20.5	21.7	23.8	25.5
CCDC number	2466026	2466030	2466036	2516032	2516034
Radiation (Å)	0.4099, synchrotron				
a (Å)	18.503(8)	18.413(8)	18.343(8)	18.215(8)	18.113(8)
b (Å)	5.50230(10)	5.48940(10)	5.47310(10)	5.45380(10)	5.43580(10)
c (Å)	10.8147(3)	10.7892(3)	10.7577(3)	10.7209(3)	10.6864(3)
β (°)	116.574(12)	116.728(12)	116.872(12)	117.160(12)	117.338(11)
V (Å^3)	984.7(5)	974.0(4)	963.4(4)	947.6(4)	934.6(4)
ρ_{calc} (g cm^{-3})	1.844	1.864	1.885	1.916	1.943
Z, Z'	6, 1.5				
Reflections measured,<br>independent,<br>observed [I > 2σ(I)]	1916<br>980<br>829	1897<br>980<br>816	1627<br>899<br>756	1883<br>951<br>807	1689<br>903<br>759
θ range (°)	2.198 / 20.210	2.202 / 20.257	2.208 / 20.289	2.215 / 20.357	2.222 / 20.422
Data completeness<br>θ_{full} = 14.237 / θ_{max}	0.315 / 0.198	0.321 / 0.199	0.299 / 0.184	0.325 / 0.196	0.313/0.187
R$_{int}$	0.0188	0.0132	0.0113	0.0199	0.0126
R1 [F^2 > 2σ(F^2)],<br>wR2 (F^2),<br>GooF	0.0477<br>0.1295<br>1.002	0.0490<br>0.1337<br>1.031	0.0520<br>0.1430<br>1.001	0.0509<br>0.1414<br>1.015	0.0525<br>0.1528<br>1.042
Parameters / restraints	190 / 0	190 / 0	190 / 0	190 / 0	190/0
ρ$_{max}$ / ρ$_{min}$ (e Å^{-3})	0.209 / -0.155	0.242 / -0.151	0.437 / -0.260	0.224 / -0.155	0.224/-0.156

Table S2 continuation.

Pressure (GPa)	27.1	29.2	31.2	34.8	37.0
CCDC number	2516036	2516038	2516035	2516037	2516033
Radiation (Å)	0.4099, synchrotron				
a (Å)	18.025(8)	17.922(8)	17.835(10)	17.673(15)	17.488(9)
b (Å)	5.41910(10)	5.40130(10)	5.38300(10)	5.3529(2)	5.35470(10)
c (Å)	10.6560(3)	10.6224(3)	10.5912(4)	10.5293(6)	10.5175(3)
β (°)	117.489(13)	117.694(12)	117.751(14)	117.94(2)	118.373(14)
V (Å^3)	923.4(4)	910.5(4)	899.9(5)	880.0(8)	866.6(5)
ρ_{calc} (g cm^{-3})	1.967	1.994	2.018	2.063	2.095
Z, Z'	6, 1.5				
Reflections measured,<br>independent,<br>observed [I > 2σ(I)]	1798<br>904<br>757	1816<br>911<br>779	1777<br>895<br>746	1699<br>860<br>701	1615<br>865<br>723
θ range (°)	2.228 / 20.480	2.234 / 20.542	2.241 / 20.607	2.254 / 20.561	2.256 / 20.721
Data completeness<br>θ_{full} = 14.237 / θ_{max}	0.311 / 0.188	0.322 / 0.191	0.319 / 0.188	0.312 / 0.185	0.295 / 0.185
R$_{int}$	0.0201	0.0241	0.0217	0.0245	0.0148
R1 [F^2 > 2σ(F^2)],<br>wR2 (F^2),<br>GooF	0.0477<br>0.1279<br>1.037	0.0561<br>0.1538<br>1.028	0.0575<br>0.1446<br>1.048	0.0645<br>0.1588<br>1.028	0.0635<br>0.1649<br>1.043
Parameters / restraints	190 / 0	190 / 0	190 / 0	190 / 0	190 / 0
ρ$_{max}$ / ρ$_{min}$ (e Å^{-3})	0.203 / -0.145	0.213 / -0.187	0.229 / -0.190	0.208 / -0.258	0.224 / -0.156

Table S3: Table on the collection and refinement data of the structures refined without satellite reflections.

Pressure (GPa)	14.0	14.8	15.6
Radiation (Å)		0.4099, synchrotron	
a (Å)	6.297(2)	6.273(2)	6.247(2)
b (Å)	5.56380(10)	5.55500(10)	5.54180(10)
c (Å)	10.953(3)	10.927(3)	10.896(3)
β (°)	115.21(3)	115.58(3)	115.8(3)
V (Å^3)	347.19(17)	343.4(4)	339.38(18)
ρ_{calc} (g cm^{-3})	1.743	1.763	1.783
Z, Z'		2, 0.5	
Reflections measured, independent, observed [I > 2σ(I)]	599 / 331 / 292	633 / 338 / 297	629 / 332 / 292
θ range (°)	2.421 / 19.845	2.427 / 19.888	2.435 / 19.943
Data completeness θ_{full} = 14.237 / θ_{max}	0.296 / 0.201	0.313 / 0.205	0.319 / 0.202
R_{int}	0.0070	0.0089	0.0085
R1 [$F^2 > 2\sigma(F^2)$], wR2 (F^2), GooF	0.0682 / 0.1813 / 1.079	0.0740 / 0.1931 / 1.118	0.0772 / 0.2122 / 1.128
Parameters / restraints	64 / 0	64 / 0	64 / 0
ρ_{max} / ρ_{min} (e Å^{-3})	0.1 / -0.192	0.218 / -0.234	0.235 / -0.223
